# MicroRNAs: Important Players in Breast Cancer Angiogenesis and Therapeutic Targets

**DOI:** 10.3389/fmolb.2021.764025

**Published:** 2021-10-26

**Authors:** Bashdar Mahmud Hussen, Sara Tharwat Abdullah, Mohammed Fatih Rasul, Abbas Salihi, Soudeh Ghafouri-Fard, Hazha Jamal Hidayat, Mohammad Taheri

**Affiliations:** ^1^ Department of Pharmacognosy, College of Pharmacy, Hawler Medical University, Erbil, Iraq; ^2^ Department of Pharmacology and Toxicology, College of Pharmacy, Hawler Medical University, Erbil, Iraq; ^3^ Department of Medical Analysis, Faculty of Science, Tishk International University-Erbil, Erbil, Iraq; ^4^ Department of Biology, College of Science, Salahaddin University-Erbil, Erbil, Iraq; ^5^ Center of Research and Strategic Studies, Lebanese French University, Erbil, Iraq; ^6^ Department of Medical Genetics, School of Medicine, Shahid Beheshti University of Medical Sciences, Tehran, Iran; ^7^ Department of Biology, College of Education, Salahaddin University-Erbil, Erbil, Iraq; ^8^ Skull Base Research Center, Loghman Hakim Hospital, Shahid Beheshti University of Medical Sciences, Tehran, Iran; ^9^ Institute of Human Genetics, Jena University Hospital, Jena, Germany

**Keywords:** breast cancer, microRNA, vascular endothelial growth factor, angiogenesis, therapeutic target 3

## Abstract

The high incidence of breast cancer (BC) is linked to metastasis, facilitated by tumor angiogenesis. MicroRNAs (miRNAs or miRs) are small non-coding RNA molecules that have an essential role in gene expression and are significantly linked to the tumor development and angiogenesis process in different types of cancer, including BC. There’s increasing evidence showed that various miRNAs play a significant role in disease processes; specifically, they are observed and over-expressed in a wide range of diseases linked to the angiogenesis process. However, more studies are required to reach the best findings and identify the link among miRNA expression, angiogenic pathways, and immune response-related genes to find new therapeutic targets. Here, we summarized the recent updates on miRNA signatures and their cellular targets in the development of breast tumor angiogenetic and discussed the strategies associated with miRNA-based therapeutic targets as anti-angiogenic response.

## Introduction

**FIGURE 1:**
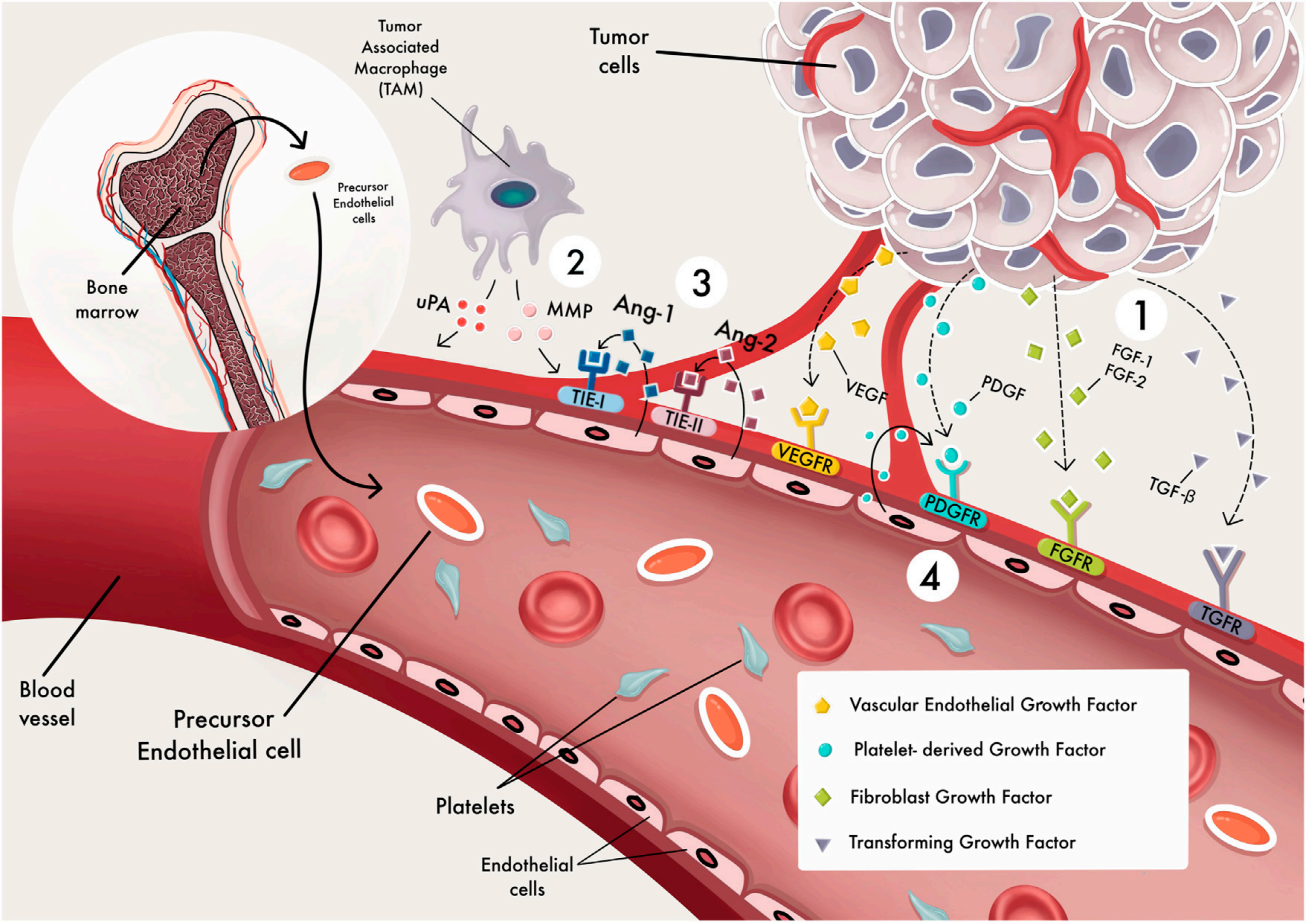
angiogenesis processes. The different stages of blood vessel development (Siegel et al., 2021). angiogenesis factors activate signal transduction pathways by binding to endothelial cells (EC) receptors (Chaffer and Weinberg, 2011). MMPs degrade the extracellular matrix, allowing ECs to migrate and proliferate outside the pre-existing capillary wall (Folkman, 1971). Endothelial cells express Tie-2 receptors for binding with Angiopoietin-1 (Ange-l); this might promote vessel sprouting, pericyte acquisition, vessel survival, and stabilization (Leone et al., 2019). ECs secrete PDGF, which attracts pericyte precursors as a chemoattractant. These cells attach to endothelial cells and grow into pericytes.

**FIGURE 2:**
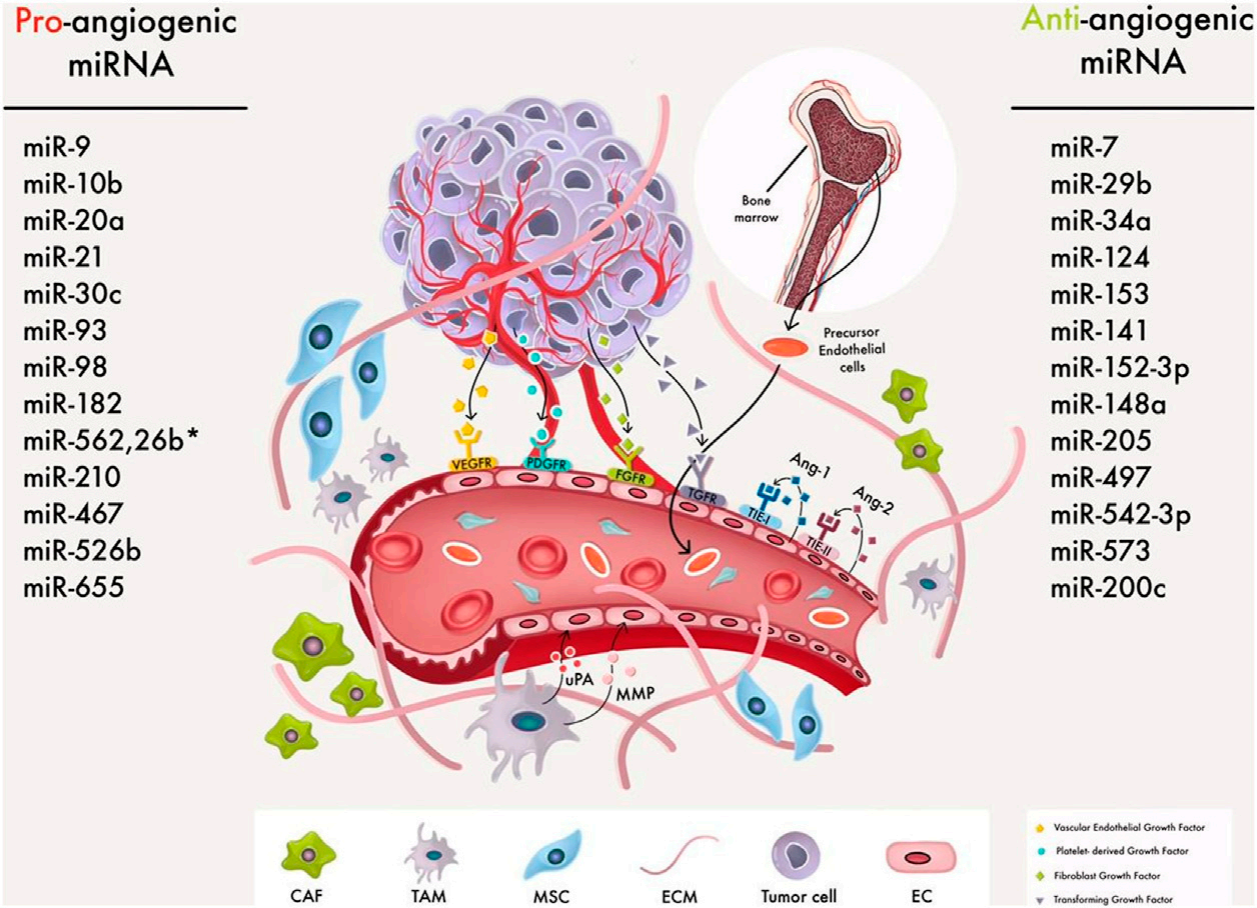
MiRNAs that regulate angiogenesis in BC cells. An anti-angiogenic miRNA can either slow or promote angiogenesis by affecting the many growth pathways involved in cancer management. For example, angiogenesis is a critical stage in tumor metastasis that might aid tumor spread, and angiogenesis in breast cancer involves TME and cancer cells communication. So miRs regulate BC angiogenesis via macrophages, MSCs, ECM, and CAFs.

Breast cancer (BC) is one of the more prevalent occurring forms of cancer in females and the second most frequently occurring type of cancer worldwide (Siegel et al., 2021), and 90 percent of breast cancer deaths are due to the formation of distant organ metastases (Chaffer and Weinberg, 2011). In breast cancer, angiogenesis, or the development of new blood vessels, is essential for both local tumor growth and distant metastasis (Folkman, 1971). Angiogenesis is a multi-step complicated process characterized by 1) MMP damages the basement membranes of tissues on a local basis, causing degradation and hypoxia immediately, 2) endothelial cells migrate in response to angiogenic factors, 3) proliferation and stabilization of endothelial cells, and 4) angiogenic factors still influence the angiogenic mechanism. The development of new vessels is regulated by a concerted action of multiple cytokines and growth factors such as anti-angiogenic and proangiogenic factors (Figure 1). miRNAs regulate tumor angiogenesis in two ways: They can inhibit or promote it (Leone et al., 2019) (Figure 2). Victor Ambros’ lab was the first to publish an article regarding miRNAs (Lee et al., 1993), which have a length of 21–25 nt, are known as short non-coding RNAs (short-RNAs) and regulate the expression of a variety of cellular proteins by modulating their messenger RNA levels (Lewis et al., 2005). They regulate gene expression in health and disease cells by binding to the 3′-untranslated region (UTR) or other regions such as the 5′ UTR, gene promoters, and the coding sequence (Broughton et al., 2016). Also, it has been established that crosstalk exists between long non-coding RNAs and miRNAs, where these two networks interact and form complex networks in gene regulation pathways (Ghafouri-Fard et al., 2021a; Ghafouri-Fard et al., 2021b; Dastmalchi et al., 2021).

According to TargetScan studies, miRNAs regulate one-third of all human genes (Lewis et al., 2005) and are essential regulators of a variety of processes, including cancer-related developments like angiogenesis (Goradel et al., 2019), metastasis (Martello et al., 2010), and drug resistance (Taheri et al., 2021). For example, global miRNA depletion inhibits the angiogenic process because miRs control the angiogenesis process (Chen et al., 2014). In addition, several investigations have described miRNA signatures in clinical breast specimens and cell lines. In particular, miRNAs can influence angiogenesis directly by affecting endothelial cell function or indirectly by changing the production of proteins that prevent or promote angiogenesis (Wang et al., 2018a). Consequently, miRNAs have attracted attention as potential targets for new anti-angiogenic therapies.

In this review, we summarized the recent updates on miRNA signatures and their cellular targets in the development of breast tumor angiogenetic and discussed the strategies associated with miRNA-based therapeutic potential as anti-angiogenic response.

In the current review, the key questions attempted to be answered were “What are the miRNA’s contribution to breast cancer angiogenesis?”, “How can miRNAs contribute to BC angiogenesis? and “What are miRNA-based therapies for angiogenesis in BC?.”

“The terms “microRNA” or “miRNA,” “breast cancer,” “therapeutic target,” and “angiogenesis” were searched in PubMed and Google Scholar. To determine whether the retrieved papers were relevant to the subject, we evaluated the abstracts of all articles. Then, all related papers (*in vitro*, *in vivo*, and human-based research) to the subject were selected to be included in the study.

## Biogenesis of microRNA

**FIGURE 3:**
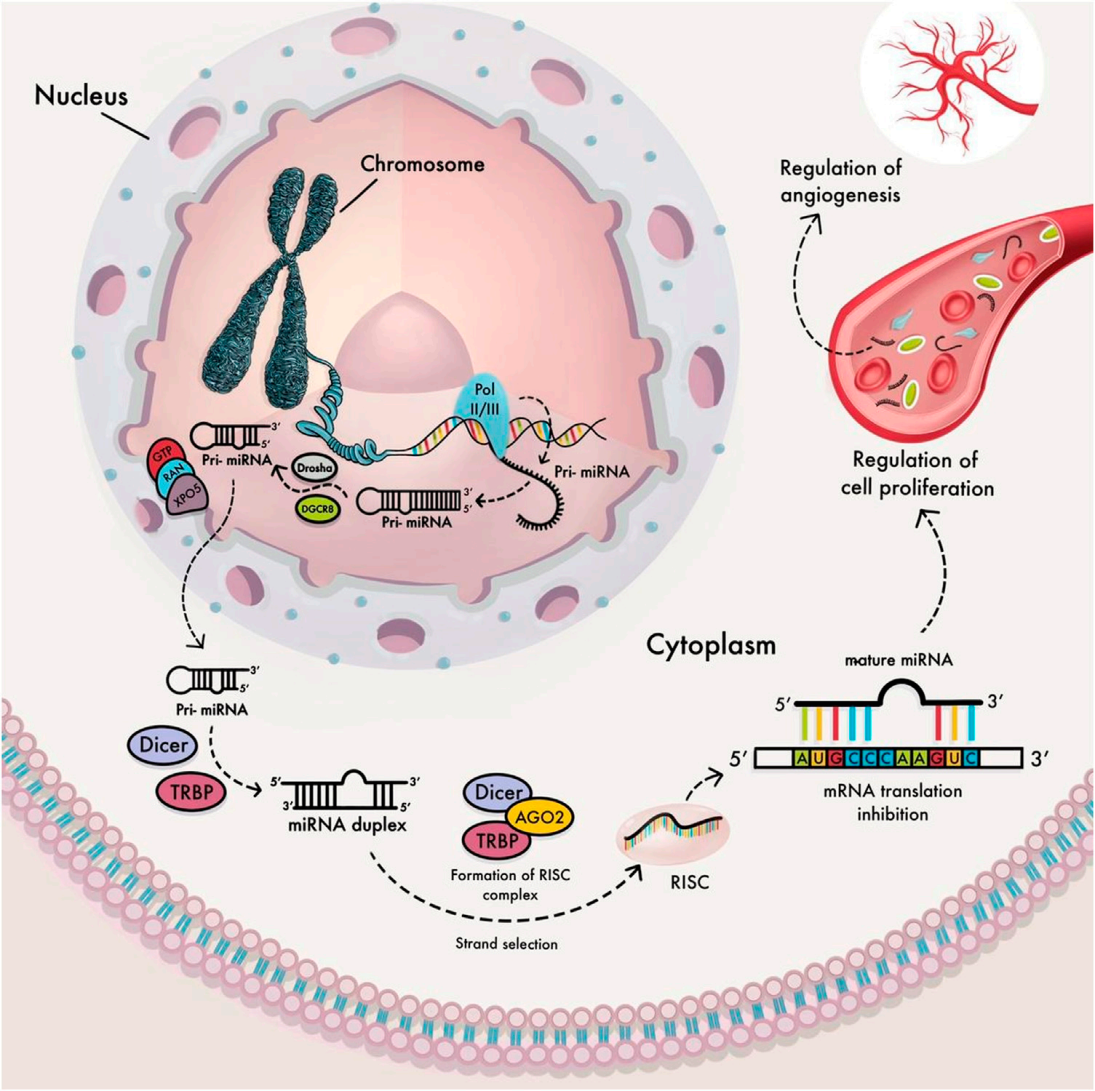
miRNA biogenesis pathway. In the nucleus, RNA polymerase II transcripts miRNA genes to produce primary miRNA transcript (pri-miRNA), then by the microprocessor complex Drosha-DGCR8 cleaved into precursor miRNA (pre-miRNA). After that, Exportin-5 transports the pre-miRNA into the cytoplasm, where it is processed into a miRNA duplex by Dicer and its interaction protein, TRBP. Once the miRNA duplex has been unwinding, it is divided into two single-stranded miRNAs for further processing. Mature miRNA is attached to the RNA-induced silencing complex (RISC) and mediates mRNA degradation or repression of protein translation within the cytoplasm. Mature active miRNAs inter to cell proliferation regulation by controlling the angiogenesis process in the tumor cells.

MiRNAs are small, single-stranded non-coding RNAs that come from pri-miRNA, which is an early-stage transcript produced by RNA polymerase II (Zeng and Cullen, 2006). The pri-miRNAs are defined by the presence of one or many incomplete hairpin structures with a stem of about 33 base pairs (Bartel, 2004). Drosha and Dicer, two RNase III family ribonucleases, process the pri-miRNA precursor in two steps (Kuehbacher et al., 2008). First, Drosha cleaves the pri-miRNA in the nucleus to create a pre-miRNA of roughly 70 nucleotides in length transported to the cytoplasm through an exportin-5 (XPO5) process (Bohnsack et al., 2004; Wu et al., 2018). Then, Dicer converts the pre-miRNA into a mature, functional, double-stranded (ds) miRNA (Carthew and Sontheimer, 2009). The mature miRNA is then covalently coupled to RISC, a multiprotein complex that includes the AGO protein, necessary for RNA silencing. Through Watson-Crick base pairing, RISC utilizes the leading strand to target the mRNA complementary, and the other strand is eliminated (Gregory et al., 2005). The miRNA binding to a 3′-UTR causes mRNA destruction or translational inhibition. The level of mRNA degradation or translational repression is determined by the degree of miRNA complementarity to the 3′-UTR. In addition, RISC can target and trigger 5′-UTR mRNA translation (Vasudevan et al., 2007). Figure 3 shows the biogenesis of miRNA.

## Tumor Angiogenesis

Angiogenesis is a multi-step and complex process to form new blood vessels from pre-existing ones. It begins with the stimulating, migrating, proliferating, and differentiating endothelial cells in response to signals from the surrounding tissue, such as hypoxia (low oxygen levels) (Bentley and Chakravartula, 2017). Each step is progressed and controlled by several pro-and anti-angiogenic factors.

VEGF, PDGF, and FGF promote new blood vessel development. VEGF-A, VEGF-B, VEGF-C, and VEGF-D are the primary forms of VEGF, and they bind to VEGF tyrosine kinase receptors (VEGFR-1-2-3) to regulate angiogenesis (Holmes and Zachary, 2005). Additionally, angiopoietins have a role in controlling endothelial cell signaling pathways. They associate with other angiogenic factors to bind with Tie-2 tyrosine kinase receptors and help the formation of endothelial tubes.

Despite the angiogenic factors, the body also produces endogenous anti-angiogenic substances such as TSP1 (Good et al., 1990), proteolytic fragments of basement membrane or extracellular matrix that comprises an inhibitor of blood vessel formation, an anti-angiogenic factor. Another anti-angiogenic factor is Endostatin, a proteolytic fragment of collagen XVIII (O'Reilly et al., 1997). The last two anti-angiogenic factors are canstatin (Magnon et al., 2005) and tumstatin (Maeshima et al., 2002), cleavage fragments of collagen IV. Furthermore, the body also produces endogenous anti-angiogenic soluble substances like IFN- α and IFN-β and angiostatin, a proteolytic fragment of plasmin (Baeriswyl and Christofori, 2009).

**FIGURE 4:**
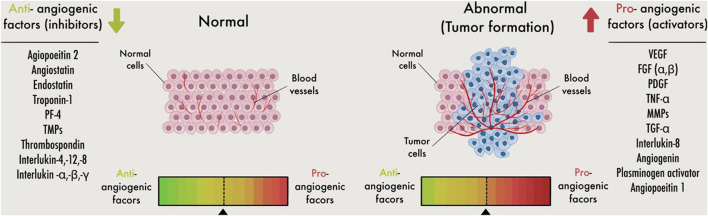
Schematic image for the angiogenic switch. Angiogenesis is controlled by a careful balance between pro-angiogenic and anti-angiogenic molecules in the bloodstream. Inadequate angiogenesis leads to a defect in the angiogenic balance, with a subsequent angiogenesis change toward excessive or reduced angiogenesis. Pro-angiogenic supports to stimulate the production of endothelial cells (ECs)during angiogenesis. Whereas anti-angiogenic factors, on the other hand, inhibit EC activation. More angiogenic than anti-angiogenic factors are involved when the angiogenic switch is activated.

The balance between pro- and anti-angiogenic factors together with differential expression, release, or activation of the numerous factors control new blood vessel formation under pathological or physiological situations (Figure 4). Under physiological conditions, stromal and endothelial cells and released chemicals constitute a dynamic system that constantly alters and produces anti-angiogenic substances that keep the vasculature quiet. First, the “angiogenic switch” is turned on because proangiogenic factors are overabundant within tumor cells, and angiogenesis occurs as a response. Then, tumor cells and invading inflammatory/immune cells can release proangiogenic factors (Baeriswyl and Christofori, 2009) (Figure 1).

A key feature of tumor development is the ability of the tumor to generate its blood supply. It is an essential step in tumor initiation, which can vary based on the characteristics of the tumor and its microenvironment (Carmeliet and Jain, 2000). In addition, this tumor vasculature facilitates the tumor’s ability to obtain appropriate oxygen, nutrition, and waste removal. Moreover, neovascularization has the potential to create an “escape route” for tumor cells to enter the circulation, allowing them to disseminate and metastasis (Aguilar-Cazares et al., 2019).

Physiological angiogenesis and tumor angiogenesis are significantly different. First, tumor angiogenesis (the formation of new blood vessels to nourish the tumor) is a long-term process. Secondly, tumor blood vessels are disorganized, chaotic vascular networks, irregular in shape, dilated, hyper permeable, and not made up of distinctive arterioles, venules, and capillaries like normal vascular systems (Siemann, 2011). Additional aspects of tumor angiogenesis, tumor cells can undergo a process known as vasculogenic mimicry, during which they alter their gene expression to look like endothelial cells (Luo et al., 2020). Vasculogenic mimicry may be induced by hypoxia due to mass tumor growth (Wei et al., 2021), which occurs when tumor cells are far away from blood vessels and consequently do not receive enough oxygen and nutrients. Individuals with malignancies that display the characteristics of vasculogenic mimicry have a poor prognosis (Yang et al., 2016). The microenvironment in anoxic conditions collaborates with oncogenic pathways to trigger new blood vessel formation and leads to cancer development (Dayan et al., 2008). Hypoxia-inducible factor-1 (HIF-1) is a major transcriptional factor influencing the development of hypoxia-induced conditions. HIF-1 is composed of α and β polypeptides. HIF-1α induces angiogenesis by upregulating the production of proangiogenic factors, including VEGF-A (Hashimoto and Shibasaki, 2015). von Hippel-Lindau (VHL) regulates the proteasome via ubiquitin degradation in normoxic conditions to keep HIF-1 levels low (Tanimoto et al., 2000). While hypoxia causes HIF-1α breakdown, the effect is that HIF-1α levels increase, and angiogenesis is promoted (Jin et al., 2020).

## Key microRNAs in Breast Cancer Angiogenesis

In the human genome, about 2,300 mature miRNAs have been identified (Alles et al., 2019), 10% of these have been shown to target EC activity and angiogenesis (Sun et al., 2018), like let7a, let‐7b, let‐7days, miR‐16, miR‐9, miR‐21, miR‐20, miR‐23a, miR‐34a, miR‐29, miR‐99a, miR‐93, miR‐100, miR‐124, miR‐125‐5p, miR‐126, miR‐146, miR‐135a, miR‐195, miR‐276, miR‐181a, miR‐181b, miR‐199b, miR‐204, miR‐200b, miR‐221 and miR‐222, miR‐296, miR‐320, miR‐361‐5p, miR‐874. Moreover, they are upregulated in ECs and linked to angiogenesis control (Poliseno et al., 2006; Kuehbacher et al., 2007; Suárez et al., 2007; Caporali and Emanueli, 2011). Thus, miRNAs are divided into two categories based on miRNA expression and angiogenesis function: 1) miRNAs that control the angiogenesis process primarily through well-characterized target genes, and 2) miRNAs controlled by pro- or anti-angiogenic factors or hypoxia (Caporali and Emanueli, 2011).

### Anti-angiogenic microRNAs in Breast Cancer

The expression of miRNAs, which regulate oncogenes and tumor suppressor genes, is changed in different types of human cancers (Hussen et al., 2021a). Therefore, these molecules have been termed “oncomiRs.” MiRNAs that are directing the process of angiogenesis are called angiomiRs, as they govern angiogenic processes in pathological and physiological circumstances (Table 1) (Suárez and Sessa, 2009). Overexpression of miRNAs is usually associated with a poor prognosis, chemotherapy resistance, and low survival (Babashah and Soleimani, 2011; Bitaraf et al., 2020). Various research groups have discovered many different miRNA expression patterns and individual miRNAs in breast cancer patients, and they have been linked to angiogenesis, invasion, and prognosis.

**TABLE 1:** miRNAs that inhibit angiogenesis in Breast cancer (ANTs: adjacent non-cancerous tissues).

miRNA	Numbers of clinical samples	Assessed cell line	Animal model	Targets/Regulators	Signaling Pathways	Association with patients’ outcome	Function	References
miR-126-3p	—	MDA-MB-231, HCC193, MCF-10A	—	RGS3	G-protein signaling 3	—	miR126-3p serves as a tumor suppressor via controlling RGS3, an essential regulator of TNBC.	Hong et al. (2019b)
miR- 126	—	MCF7	—	VEGF-A	—	—	VEGF-A is downregulated due to the expression of miR-126 in breast cancer	Alhasan, (2019)
miR-126-3p	—	MCF7	Rat	VEGFA and PIK3R2	VEGF/PI3K/AKT	—	The expression of miR-126 has been shown to regulate the VEGF/PI3K/AKT signaling pathway	Zhu et al. (2011)
miR-206	—	MDA-MB-231	—	*VEGF*, *MAPK3*, and *SOX9*	—	—	miR-206 downregulated in TNBC through targeting *VEGF*, *MAPK3*, and *SOX9*	Liang et al. (2016)
		MCF-7						
miR-206	50 formalin-fixed and paraffin-embedded BC tissue	MDA-MB-231, SKBR3, MDA-MB-1739, HCC70, MDA-MB-361	—	VEGF MAPK3	—	—	In TNBC cells, miR-206 controls VEGF and MAPK3 expression	Salgado et al. (2018)
		MCF-7						
miR-4500	—	MCF10A, MCF7, BT474, MDA-MB-231, MDA-MB-468	Nude mice	RRM2	MAPK	tumor suppressor in BC	miRNA-4500 suppresses MAPK signaling via regulating RRM2	Li et al. (2020a)
miR-497	45 pairs of clinical samples and ANTs	MCF-7, T-74D, MDA-MB-468, MDA-MB-453, MDA-MB-435	—	HIF-1α	—	suppresses the proliferation and tube formation	miRNA-497 suppresses BC angiogenesis by direct targeting HIF-1α	Wu et al. (2016)
miR-100	Six healthy persons	MCF-7, MDA-MB-231, and T47D	—	mTOR	mTOR/HIF1α/VEGF	—	miR-100 regulates neovascularization via mTOR.	Pakravan et al. (2017)
miR-153	—	HEK293T	13 Nude mice	HIF1α	IRE1α-XBP1	—	Hypoxia promotes miRNA-153 to fine-tune HIF-1α/VEGFA-stimulated angiogenesis in BC.	Liang et al. (2018a)
		MCF10A						
		MDA-MB-231						
		HCC1937						
miR-190	12 pairs of tumors and ANTs	MCF‐7, MCF‐10A, T47D, MDA‐MB‐231, Bcap37	36 Nude mice	STC2	AKT-ERK	Overexpression of miRNA‐190 leads to inhibition of tumor growth in BC.	Angiogenesis in BC is inhibited by miR190, which targets STC2	Sun et al. (2019)
miR-200 family	—	MDA-MB-231, T47D, H578T, BT-549, MCF-7	Nude mice	IL-8 and CXCL1	—	—	Through targeting interleukin-8 and CXCL1, miRNA-200 inhibits angiogenesis	Pecot et al. (2013)
miR-145	—	MDA-MB-231	Nude mice	IRS1	PI3K/Akt and Ras/Raf/MAPK	-	miR-145 inhibits angiogenesis by inhibiting vascular endothelial cell tube formation	Pan et al. (2021)
miR- 148a-3p		MDA-MB-231	Nude mice	IGF-1R and VEGF	—	—	Angiogenic factors, such as VEGF and IGF-1R, are targeted by miR-148a-3p, which decreases the expression of these factors	Lacerda et al. (2019)
miR-148a	—	Hs-578T, MDA-MB-231	—	Wnt-3′- UTRs	Wnt/β-catenin signaling pathway	—	miR-148a inhibits angiogenesis by suppressing GLA via the Wnt/-catenin pathway	Mu et al. (2017)
miR-148a	68 breast tissues and 22 pairs of tumors and ANTs	MCF-7, T47D, MDA-MB-231	—	IGF-IR and IRS1	AKT and MAPK/ERK signaling pathways	—	miR-148a inhibits tumor growth by binding to IGF-IR and IRS1	Xu et al. (2012)
miR-152	—	MDA-MB-468	Nude mice	IGF-IR	—	—	miRNA-152 suppresses HIF-1 and VEGF via modulating IGF-IR and IRS1	Marques et al. (2018)
				IRS1				
miR-153	Seven pairs of tumors and ANTs	MCF7, MDA-MB-231, and HCC1937	—	ANG1	—	—	miR-153 reduced endothelial cell migration and tube formation by targeting ANG1in breast cancer	Liang et al. (2018b)
miR-139	—	HCC180, MCF-10A	48	SOX8	—	—	miR-139 reduced TNBC cell growth by targeting SOX8	Dong et al. (2020)
		Six and BT549	Nude mice					
miR-10a	—	MCF-7, MCF-10A and MDA-MB-231	—	p-Akt	PI3K/Akt/mTOR	—	miRNA-10a inhibited phosphoinositide/Akt/mTOR signaling in breast cancer cells	Ke and Lou, (2017)
				p-mTOR, p-p70S6K, PIK3CA				
				Cyt C				
miR-195	—	MDA-MB-231, MCF7, MDA-MB-435, MDA-MB-453, SK-BR3	—	IRS1	—	—	miR-195 reduces tumor angiogenesis by inhibiting the IRS1-VEGF axis	Wang et al. (2016)
		T47D, ZR-75-30						
miR-19b-1	—	MDA-MB-231	Nude mice	VEZF1	—	—	miR-19b-1 can activate VEGFR to decrease angiogenesis in BC.	Yin et al. (2018)
		MCF-7		VEGF				
miR-199b-5p	-	T47D, MCF-7, and BT474	Nude mic	ALK1	ALK1/Smad/Id1	—	miR-199b-5p targets ALK1 to suppress angiogenesis in BC.	Lin et al. (2019)
miR-542-3p	72 breast cancer patients	HMECs, SVEC4-10, 4T1 and HEK293T	—	Ang2	—	With the late stages of BC, miRNA-542-3p could be useful for tracking disease progression	Breast cancer miR-542-3p targets Angiopoietin-2 to decrease tumor angiogenesis	He et al. (2014)
miR-542-3p	52 breast cancer patients	MDA-MB-231 and HEK293T	Nude mice	Angpt2	—	Lower miR-542-expression was linked to increased ANG expression.3p expression	Breast cancer miR-542-3p targets angiopoietin-2 to decrease tumor angiogenesis	He et al. (2015)
miR-497	45 pairs of tumors and ANTs	MCF-7	BALB/c nu/nu mice	HIF-1α and VEGF	miR-497/HIF-1α pathway	—	miR-497 overexpression reduced the expression of HIF-1 and VEGF.	Wu et al. (2016)
		T-74D, MDA-MB-453, MDA-MB-435						
		MDA-MB-468						
miR-519c	34 breast cancer tissues	MCF-7, SKBR3, MDA-MD431, MDA-MB231, T47D	BALB/c Nude mice	HIF-1α	HGF/c-Met signaling pathway	—	miR-519c controls angiogenesis by inhibiting HIF-1 *in vitro* and *in vivo*	Cha et al. (2010)
miR-573 and miR-578	43 FFPE	HEK293, MCF-7 and SUM149PT	—	VEGFA and ANGPT2	VEGF, FAK, and HIF-1 signaling pathways	—	miR-573 and miRNA-578 can control angiogenic markers like HIF-1, VEGF, and focal adhesion kinase	Danza et al. (2015)
miR-29b	—	MDA-MB-231	Nude	Akt3	—	—	miR-29b slows tumor development by targeting Akt3 and decreasing angiogenesis	Li et al. (2017)
miR-199b-5p	—	MDA-MB-231		ALK1	ALK1/Smad/Id1	—	miR-199b-5p overexpression decreased tumor development and angiogenesis	Lin et al. (2019)
miR-204	58 breast cancer samples	MCF-7, MDA-MB-231, ZR-75, MDA-MB-453, T457-D	Nude mice	FOXC1, RAB22A, SMAD4	—	Deregulated expression of microRNAs has been associated with angiogenesis	miR-204 reduces angiogenesis in BC cells by targeting FOXC1, RAB22A, and SMAD4	Flores-Pérez et al. (2016)
miR-204	—	MCF-7, MDA-MB-231, T457-D, ZR-75, MDA-MB-453	Athymic nu/nu mice	ANGPT1 and TGFβR2 genes	angiopoietin signaling	—	miR-204 targets proangiogenic genes (ANGPT1 and TGF-R2) to decrease cell proliferation, invasion, and angiogenesis	Flores-Pérez et al. (2016)

### microRNA-126

miRNA-126 is expressed mainly in ECs, and it is significantly linked with angiogenesis throughout normal development and wound healing (Harris et al., 2008). The two mature strands of the pre-miR-126 are miR-126-3p and miR-126-5p, which have distinct cell-type and strand-specific functions in angiogenesis (Zhou et al., 2016).

MiRNA-26, which targets both VEGFA and PIK3R2, plays a significant role in angiogenesis in human breast cancer, and its expression was decreased (Zhu et al., 2011). In addition, Overexpression of miR-26a in MCF7 cells has been found to reduce cancer development, tumor angiogenesis and induce apoptosis by inhibiting VEGF/PI3K/AKT signaling pathway (Fish et al., 2008; Zhu et al., 2011). Furthermore, ectopic expression of miR-126 in BC cells reduced CD97, a G-coupled receptor that promotes cell invasion and angiogenesis via integrin signaling. (Lu et al., 2014). Another study found that miR-126 controls metastasis and angiogenesis through targeting the pro-angiogenic genes (IGFBP2, PITPNC1, and c-Mer kinase) (Png et al., 2011). These findings suggest that a single miRNA (miR-126) regulates cell survival and angiogenesis, with the possibility to help control vascular function and development.

### microR-497

According to the miRNA growing evidence, miR-497 has been found that regulate the proliferation, migration, and survival of BC cells (Liu et al., 2016). In a mouse xenograft model, miR-497 inhibits tumor development and endothelial cell tube formation (Wu et al., 2016). Furthermore, it was shown that the levels of VEGF and HIF-1 protein were decreased due to the overexpression of miR-497 (Wu et al., 2016). Tu and his group (Tu et al., 2015) described that increasing levels of miR-497 in 4T1 cells suppressed the growth of BC cells, angiogenesis, and VEGFR2 expression when injected in transgenic mice with VEGFR2-luc. Further, it has been recommended (Wu et al., 2016) that miR-497 may serve as a novel treatment approach for BC via inhibiting proangiogenic molecules (HIF-1α and VEGF).

### microR-155

MiR-155 expression is considerably increased in BC and is highly associated with high tumor grade, progressing stage, and lymph node, but is negatively associated with cancer survival (Chen et al., 2012). Tumors respond to hypoxia by activating a genomic pathway, including miRNAs dependent on HIF1α and the hypoxia-induced pathway. Recent data showed that miR-155 has a crucial role in HIF1α-induced angiogenesis, and its expression is differentially regulated in BC (Chang et al., 2011). In BC tissues, the comparative expression of miR-155 was substantially greater than in normal tissues. Increased levels of miR-155 were all associated with tumor grade, tumor stage, and lymph node metastasis (Chen et al., 2012). Furthermore, the levels of miR-155 are negatively related to VHL protein; an E3 ubiquitin ligase inhibits members of the HIF1 family (Kong et al., 2014). Additionally, in patients with breast cancer, an increase in the expression of miR-155 may offer a prognostic marker and therapeutic target.

### microR-542-3p

The control of Angpt2 by miRNAs has been established, indicating that miRNAs are key modulators of angiogenesis (He et al., 2014). MiR-542-3p has been shown to serve as a tumor suppressor gene and has been linked to the control of various cancers (Long et al., 2016; Wang et al., 2018b), including breast cancer (Lyu et al., 2018). The level of miR-542-3p is inversely correlated to the clinical progression of patients with advanced-stage of BC. The use of an *in-vitro* BC mice model revealed that the overexpression of miR-542-3p could suppress tumor growth, formation of tubules, and metastasis (He et al., 2015). He et al. miR-542-3p downregulates the expression of angiogenin, allowing it to be overexpressed in tumor cells and promote angiogenic activation in both *in vitro* and *in vivo* models (He et al., 2015). CEBPB and POU2F1, which are transcription factors for miR-542-3p, were shown to be suppressed by angiogenin, which might function as a new tumor-endothelial cell signal pathway (He et al., 2015). As a result, miR-542-3p presents novel targets for BC prevention and therapy.

### microR-568

The miR-568 has been found to be present in the circulation of women with breast cancer (Leidner et al., 2013). lncRNA Despite being well-known for promoting metastasis in several malignancies, such as BC, Hotair has the potential to alter the expression of gene patterns and inhibit the production of miR-568, a critical tumor suppressor gene (Li et al., 2014). Furthermore, it appears that miR-568 is causing low levels of NFAT5 expression, which in turn sustains VEGFC and S100A4 expression, both of which are angiogenic and metastatic transcriptional activators of NFAT5 (Li et al., 2014). Despite these findings, additional study is required to identify or better understand the connection between lncRNAs and miRNAs for more efficient treatment approaches.

### microR-204

miR-204 is a new multi-target anti-angiogenic miRNA that fights BC. in MCF7 BC cells, miR-204 mediates tumor-suppressing effect, and expression of miR-204 induces the inhibition of proliferation, invasion, and metastasis through targeting p-AKT and p-PI3K significantly (Fan et al., 2019). *In vivo* vascularization and angiogenesis were similarly reduced in nu/nu mice by miR-204, which is consistent with previous findings (Salinas-Vera et al., 2019). The levels of proangiogenic ANGPT1 and TGFβR2 proteins were decreased in MDA-MB-231 BC cells after treating with miR-204 (Salinas-Vera et al., 2019). Furthermore, ectopic expression of miR-204 exhibits decreasing vascular endothelial growth factor (VEGF) levels and a reduced number of brunches of capillary tubes (Salinas-Vera et al., 2018). Conclusively, multiple proteins associated with the PI3K/AKT, RAF1/MAPK, FAK/SRC, and VEGF pathways were downregulated and phosphorylated due to increased levels of miR-204 (Salinas-Vera et al., 2018; Hong et al., 2019a). This new finding reveals miR-204 has a new, yet-unproven role in regulating the crucial synergy of the PI3K/AKT/FAK mediators that are important in VM development.

### microR-29

The family of miR-29a, miR-29b, and miR-29c genes have similar structures, have a high degree of sequence similarity, and serve as a target-identifying foundation for molecules (Sun et al., 2018). miR-29b acts as a tumor suppressor, which inhibits angiogenesis and tumor development. However, in several malignancies, including endometrial carcinoma (Chen et al., 2017), HCC (Fang et al., 2011), ovarian cancer (Sugio et al., 2014), and BC (Li et al., 2017), miR‐29b levels are downregulated. miRNA-29b Expression in breast cancer impairs the development of capillary-like tubular structures in HUVECs, as well as their ability to proliferate, migrate, and stop tumor progression (Li et al., 2017). Moreover, miR-29b acts to target Akt3 and inhibit angiogenesis and tumor growth by acting as an anti-angiogenesis and anti-tumorigenesis agent through the VEGF and C-myc arrest in BC cells (Li et al., 2017). Importantly, miR‐29b might be an efficient anti-cancer treatment by way of therapeutic administration.

### microR-4500

MiR-4500 was expressed poorly in BC cell lines, and RRM2 was a target gene (Li et al., 2020a). Additionally, a high expression of miR-4500 is seen in BC cells. Its expression in BC cells impairs the MAPK signaling through control of RRM2, which decreases proliferation, invasion, and angiogenesis while causing apoptosis. (Li et al., 2020a). Based on the findings that therapeutic methods should target the elevation of miR-4500, which might be a therapeutically feasible target in breast cancer treatment, this implies that therapies should focus on raising miR-4500 levels. However, to completely understand the precise processes and mechanisms of miR-4500 in BC, further studies are required on tissues from patients and additional BC cell lines.

### microR-200

It has been determined that miR-200 can inhibit angiogenesis in breast cancer’s environment and make it a therapeutic potential substance. Pecot et al. showed that miR-200 reduces angiogenesis via targeting interleukin-8 and CXCL1, which are produced by the tumor endothelium and cancer cells, and indirectly by targeting interleukin-8 (Pecot et al., 2013).

Furthermore, miR-200 family members have been shown to regulate the formation of blood vessels and angiogenesis by suppressing VEGF signaling ((Choi et al., 2011)). *In vitro*, angiogenesis was inhibited by miR-200b, which caused activation of the Notch system, which then triggered wound healing (Qiu et al., 2021). Thus, by modulating the expression of VEGF, the miR-200 family may offer a possible anti-angiogenesis treatment for treating cancer and other illnesses dependent on angiogenesis.

### microR-190

In BC cells, miRNA-190 has the potential to target STC2 negatively, and through suppressing the AKT-ERK pathway, it has the potential to impede migration, invasion, EMT, and angiogenesis ((Sun et al., 2019)). Angiogenesis is influenced by the tumor microenvironment, which alters the cellular mechanisms required for vascular growth. MiRNA-190 suppresses the tumor microenvironment by targeting a set of angiogenic genes including RAS2, TCF4, HGF, Smad2, Smad4, IGF1, Jak2, and VEGF *in vivo* and *in vitro* (Hao et al., 2014). Furthermore, these genes, targeted by miRNA-190, have been shown to control VEGF expression (Hao et al., 2014). Moreover, it has been demonstrated that miR-190 substantially inhibits tumor metastasis, particularly angiogenesis (Hao et al., 2014). These findings collectively suggest that miR-190 is a promising anticancer target in therapeutic applications.

### microR-148a

In various kinds of cancer, including BC (Yu et al., 2011), HCC (Pan et al., 2014), and ovarian cancer (Wang et al., 2018c), miRNA-148a-3p works as a tumor suppressor that is substantially downregulated. miRNA-148b-3p has also been implicated in the control of carcinogenesis, according to recent research. Interestingly, both miRNA-148a and miRNA-148b are essential regulators of EC migration in responses to VEGF, and they are also important regulators of EC proliferation. Because miRNA-148a/b-3p targets NRP1, upregulating its expression in ECs from its normally low endogenous levels has a significant inhibitory effect on VEGF-induced activation of VEGFR2 and downstream signaling (Kim et al., 2019). HIF-1a expression, which is required to form a functional HIF-1 transcription factor, was likewise reduced by MiR-148a. HIF-1 is a transcription factor that regulates the production of VEGF and other angiogenesis regulators (Semenza, 2003). As a result, we believe that miR-148a suppresses angiogenesis in breast tumors.

Furthermore, it has been established that miR-148a overexpression inhibited the angiogenesis produced by MCF7 cells in BC by directly targets ERBB3 (Yu et al., 2011). Additionally, overexpression of miR-148a targets IGF-IR and IRS1 suppresses BC cells proliferation and tumor angiogenesis by suppressing their downstream AKT and MAPK/ERK pathways (Xu et al., 2013). Following these findings, it appears that miRNA-148a might potentially be a promising therapeutic target in cancer treatment in the upcoming years.

### microR-199b-5p

According to previous studies, miRNA-199b-5p is a tumor suppressor. It was revealed to be downregulated in various BC cell lines and reduced in VEGF-induced human umbilical vein epithelial cells (HUVECs) (Lai et al., 2018; Lin et al., 2019). Interestingly, the migration and angiogenesis of HUVECs were decreased by ectopic expression of miRNA-199b-5p, whereas inhibition of miRNA-199b-5p induced the reverse effect. Similarly, HUVECs treated with high levels of miR-199b-5p exhibited suppressed ALK1 mRNA and protein production due to direct binding to the 3′UTR of ALK1 (Lin et al., 2020). Aside from that, high levels of miR-199b-5p in HUVECs reduced the activation of the ALK1/Smad/Id1 signaling by BMP9 in BC. As a result of these findings, miR-199b-5p, which primarily affects ALK1, has been identified as an anti-angiogenic factor, suggesting that miR-199b-5p might be used as an anti-angiogenic strategy in treating cancer.

### microRNA-195

miRNA-195 is one of the genetic markers found on chromosome 17p13.1, known as the origin of intron 7. miRNA-195 has been characterized as a tumor suppressor molecule that is often dysregulated in many malignancies, such as BC (Yu et al., 2018). miR-195’s anti-cancer activity is attributed to its target molecules, FASN, ACACA, HMGCR, and IRS1, which help slow down BC cell growth, migration, angiogenesis, and metastasis. (Singh et al., 2015; Wang et al., 2016). In both BC cell lines and BC tissues, miR-195 is negatively linked to Insulin receptor substrate 1 (IRS1) (de Sales et al., 2021). IRS1 expression is downregulated by induction miR-195 using a miR-195 oligo transfection method or infection with a lentivirus containing the miR-195 gene (Wang et al., 2016; Lai et al., 2018). These findings imply that miR-195 replicas are promising BC treatment agents.

### Pro-angiogenic microRNAs in Breast Cancer

miRNAs are involved in the initiation and progression of several tumor characteristics, such as tumor invasion, angiogenesis, and metastasis. Recent investigations have suggested that several miRNAs suppress angiogenesis in the BC. These studies present novel therapy options for treating angiogenesis in BC (Table 2).

**TABLE 2:** miRNAs that promote angiogenesis in Breast cancer (ANTs: adjacent non-cancerous tissues).

miRNA	Numbers of clinical samples	Assessed cell line	Animal model	Targets/Regulators	Signaling Pathways	Association with patients’ outcome	Function	References
miR-20a	breast cancer patients (n = 108)	MCF7 MDA-MB-231	—	VEGFA	—	Promoting metastasis	miR-20a promotes aberrant vascular mesh size and excessive VEGFA expression	Luengo-Gil et al. (2018)
miR-20a/b	32 breast cancer patients	—	—	VEGF-A and HIF-1alpha	—	metastatic heterogeneity	In BC patients, VEGF-A and HIF-1alpha target proteins correlated negatively with miR-20a/b	Li et al. (2012)
	16 controls							
miR-20b	23 paired clinical breast cancer tissues and ANTs	MCF7, SK-BR-3	Nude mice	PTEN	PTEN-PI3K-Akt pathway	—	miR-20b acted as a tumor promoter by targeting PTEN expression	Zhou et al. (2014)
		T-47D						
		ZR-75-30						
miR-21	—	MVT1 and E0771	FVB/N	Col4a2, Spry1, and Timp3	CSF1-ETS2 pathway	—	miR-21 promotes tumor metastasis and angiogenesis by suppressing the CSF1-ETS2 pathway	Isanejad et al. (2016)
			Nude mice					
miR-29	79 breast cancer samples and 60 pairs of tumors	MCF-10A, MCF-7	BALB/c Nude mice	—	TET1	poorer prognosis	miR-29a stimulates BC cell proliferation and EMT through TET1	Pei et al. (2016)
		MDA-453, MDA-231						
miR-93	—	MT-1	Nude mice	LATS2	—	—	miR-93 targets LATS2 to promote angiogenesis and metastasis	Fang et al. (2012)
miR-10b	-	HMEC-1, MDA-MB-231	Nude mice	HoxD10	—	—	miR-10b targets HoxD10 mRNA to induce angiogenesis	Shen et al. (2011)
miR-655/526b	105 tumor tissue samples	MCF7 and MDAMB231	—	VEGFA, VEGFD, VEGFC, COX-2	PI3K/Akt and ERK pathways	—	miR-526b-655 induce both angiogenesis and lymph angiogenesis in BC.	Hunter et al. (2019)
	20 non-cancerous tissues			*PTEN*				
				LYVE-1				
miR-155	231 breast cancer patients	BT474	nude mice	VHL	—	poor prognosis	miR-155 stimulates tumor angiogenesis and proliferation by targeting VHL.	Kong et al. (2014)
		HS578T MDA-MB-157						
miR-9	—	BT-474, MDA-MB-231	—	LAMC2, ITGA6, and EIF4E	—	—	miR-9 targets mRNA from genes involved in VEGF expression (LAMC2, ITGA6, EIF4E)	Kim et al. (2017)
miR-10b	130 patients	—	—	HOXD10	—	breast cancer aggressive behavior, distant metastasis, and poor prognosis	miR-10b expression associated with metastases and angiogenesis in node-negative breast cancer	Liu et al. (2017)
miR-182	45 pairs of tumors and ANTs	MCF-7	—	FBXW7	HIF-1α-VEGF-A pathway	promote breast cancer progression	miR-182 promotes breast cancer angiogenesis by increasing HIF-1 expression	Chiang et al. (2016)
		EA. hy926						
		H184B5F5/M10						
miR-183-5p	50 pairs of BC tissues and neighboring non-tumor breast tissues	BT549, MCF-10A	—	FHL1	—	poor prognosis	The miR-183-5p inhibits FHL1 and hence increases tumor proliferation and angiogenesis	Li et al. (2020b)
		SK-BR-3, MDA-MB-231, MCF7, MDA-MB-453, BT20						
miR-373	196 breast cancer patients	—	—	VEGF and cyclin D1	—	miRNA-373 expression level has unfavorable prognostic factors for breast cancer	By targeting VEGF and cyclin D1, miR-373 increases angiogenesis and metastasis	Bakr et al. (2021)
	76 Benign patients							
	49 Healthy controls							
miR-210	299 paraffin-embedded breast cancer tissue	—	—	HIF-1α	—	breast cancer progression	miR-210 induced angiogenesis by targeting HIF-1α -VEGF signaling	Foekens et al. (2008)

### microR-20b

miRNA-20 is a member of the miRNA-17–92 cluster, and it has been characterized as an oncogenic miRNA molecule that is often dysregulated in many malignancies, such as BC (Li et al., 2012). miRNA-20b is a potential oncogene that affects the control of VEGF expression in MCF-7 breast cancer cells by targeting HIF-1α and STAT3 (Cascio et al., 2010). The other study found that miR-20b was elevated in human BC tissues and predicted that the anti-oncogenic PTEN gene might target miRNA-20b (Zhou et al., 2014). These data suggest that miR-20b may be used as a potential biomarker and a viable target for diagnosing and treating breast cancer.

### microR-29a

In several types of malignancies, including esophageal (Liu et al., 2015a), colon cancer (Leng et al., 2021) and BC (Rostas et al., 2014) miR-29a was shown to be upregulated. The essential process that leads to metastasis is the EMT. miR-29a, which is upregulated in many types of cancer, has been shown to promote EMT in BC (Rostas et al., 2014). Furthermore, *in vivo* and *in vitro*, miRNA-29a upregulation led to TET1 reduction, which increased cell proliferation and EMT in BC (Pei et al., 2016). Thus, it appears that miR-29a is a novel biomarker for BC detection and a possible treatment target.

### microR-9

The miRNA-9 expression is abnormal in many cancers; however, the Role of miR-9 in cancers is still debated, in some studies indicating that it is a proangiogenic oncomiR as in BC (Bertoli et al., 2015) or as a tumor suppressor such as in melanoma (Bu et al., 2017). Kim et al. recently discovered that miRNA-9, an angiogenic mediator, selectively targets mRNA from genes involved in stimulating VEGF expression (Kim et al., 2017). Their study observed that miR-9 could inhibit the production of VEGFA by binding to the products of the ITGA6 gene, which encodes α6β4 integrin complex subunit in BC cell lines (Kim et al., 2017). *In vitro*, it has already been established that the integrin (α6β4) subunit enhances the VEGF expression by activating the mTOR pathway (Soung et al., 2013). Similarly, miR-9-mediated E-cadherin increases VEGFA expression in breast cancer via activating beta-catenin signaling in animal models and cell lines (Ma et al., 2010).

Furthermore, the same study found that miRNA-9 targets the CDH1 expression, which is translated into E-cadherin protein and increases the nuclear localization and activity of β-catenin (Ma et al., 2010), which are both essential in tumorigenesis (Yu et al., 2019). Lastly, they observed that upregulation or elevated levels of miRNA-9 in breast cancers promotes angiogenesis (Ma et al., 2010). These findings provide evidence that miRNA-9 has a proangiogenic function for the development of cancer.

### microR-10b

The miR-10b genomic location is in front of the HOXD4 gene and has more attention because of its highly conservative (Tehler et al., 2011). *In vivo* and *in vitro* studies have revealed the importance of miR-10b in angiogenesis (Lin et al., 2012). It was shown that axillary lymph node-negative breast cancer patients had an increased microvessel density (MVD), which was correlated with raised miRNA10b (Liu et al., 2017). Plummer and his colleagues found that miR-10b shows increased expression and promotes stimulation of VEGF in high-grade human breast cancer (Plummer et al., 2013). The increased expression of miR-10b is considered to have a role in increasing the capacity of endothelial cells to create blood vessels by reducing the anti-angiogenic pathway gene expression (Shen et al., 2011). By binding to the 3′ UTR of HoxD10 mRNA, miRNA-10b is capable of targeting HoxD10 mRNA and inhibits the production of HoxD10 protein (Shen et al., 2011). Interestingly, FLT1 is believed to inhibit VEGF and VEGFR2 interaction from stopping the development of new blood vessels (Fong et al., 1999). It has been observed that inhibiting the expression of miR-10 in HUVECs exposed to low concentrations of VEGF decreases the VEGFR2 phosphorylation, which inhibits the VEGF-dependent angiogenesis (Hassel et al., 2012).

### microR-21

miR-21, a hypoxia-inducing miRNA, has participated in developing cancer, angiogenesis, and stimulation of VEGF signaling in patients with BC (Foekens et al., 2008). Oncogenic mi R-21, which is related to the advanced tumor stage, lymph node, and poor patient mortality, was found as a potential molecular prognosis mark for BC development (Yan et al., 2008). In a VEGFR2-Lucent mice model of BC, a miR-21 antagomir substantially decreased cancer growth and tube formation by directly inhibiting the VEGF/VEGFR2/HIF1 pathway (Zhao et al., 2013). Interestingly, this work demonstrated that miR-21 inhibition causes apoptosis in BC cells and HUVECs via upregulating the PTEN gene. This microRNA has also been shown to be effective in inhibiting angiogenesis.

Evolving angiogenesis in mice carrying BCs with a luciferase gene that activates the VEGF/VEGFR2 pathway may be stopped if a molecule called miR-21 is inhibited by blocking HIF-1α/VEGF/VEGFR2 signaling (Zhao et al., 2013). Furthermore, this work demonstrated that blocking miRNA-21 leads to PTEN overexpression, contributing to BC cell and HUVEC death. The presence of miRNA-21 has also been shown to inhibit the growth of new blood vessels. Liu et al. exposed that a BC oncogene, metadherin (MTDH), can promote angiogenesis by stimulating the miR-21/ERK-VEGF-MMP2 pathway (Liu et al., 2015b). According to recent research, exercise and hormone treatment decreased tumor growth and angiogenesis in a mouse model of invasive breast cancer by reducing levels of miR-21, ER, HIF-1, VEGF, and raising levels of miR-20 IL-10, let-7a, and PDCD4 in tumor tissue (Isanejad et al., 2016). Thus, reductions in miR-21 levels are associated with an anti-angiogenic response in breast cancer. Additionally, miR-21 can enhance tumor metastasis and angiogenesis by inhibiting anti-angiogenic genes such as TIMP3, COl4a2, and Spry1 in tumor-infiltrating myeloid cells (Isanejad et al., 2016).

### microR-93

It is located on the 7th chromosome and is part of the miRNA-106b-25 cluster. miR-93 is one of the miRNAs frequently found to be overexpressed in tumors (Sun et al., 2018). Previous research has discovered that miRNA-93 is increased in BC and that it functions as an oncomiR, promoting angiogenesis. (Fang et al., 2012; Liang et al., 2017; Sun et al., 2018). Fang and his team found that miRNA-93 is required to promote angiogenesis, enhanced EC proliferation and migration, and tube formation (Fang et al., 2012). Also, miRNA-93 is upregulated in breast cancer, stimulating new blood vessel growth by blocking the homology 2 gene (LATS2) (Fang et al., 2012). MiR-93, on the other hand, appears to have a function in the inhibition of angiogenesis in some pathological conditions (Fabbri et al., 2016). According to the study by Liang et al., TNBC specimens with greater levels of miR-93-5p had increased blood vessel density. They also revealed that overexpressing miRNA-93-5p in HUVECs enhanced proliferation, migration, and cell sprouting *in vitro*, but inhibiting miRNA-93-5p reduced migration and angiogenic ability (Liang et al., 2017). miR-93 is involved in tumor angiogenesis by inhibiting several targets, particularly VEGF, EPLIN, integrin-β8, IL-8, and LATS2 in TNBC tissues (Liang et al., 2017). Fang et al. have demonstrated that tumor xenografts formed from breast cancer cell lines transfected with miR-93 showed increased vascular density and metastatic ability and a greater capacity for lung metastasis than tumors transfected with a vector without miR-93 (Fang et al., 2012).

Additionally, they found that miR-93 might promote invasion and tumor angiogenesis by silencing LATS2 expression. Furthermore, miR-93, an oxygen-responsive microRNA, might disrupt NCOA3, an epigenetic factor that mediated tumor suppression and inhibits cGAS-mediated antitumor immunity in breast cancer (Wu et al., 2017). Due to this, tumor angiogenesis may be promoted by the hypoxia-regulated miRNAs like miR-93, which also participates in immunosuppression. Altogether, miRNA-93 has a dual impact on angiogenesis in various human tissues and cells, and these effects are mediated through a variety of molecular pathways.

### microR-182

The miRNA-182-183-96 cluster contains miR-182, which is located on chromosome 7q32. Overexpression of miR-182 has been found in BC cells, and this miRNA inhibits FOXO1, MTSS1, MIM, and BRCA1 and, therefore, negatively impacts cell proliferation angiogenesis and DNA damage response (Guttilla and White, 2009; Lei et al., 2014). Furthermore, increasing miR-182 expression leads to an increase in HIF-1α and VEGFA activation by direct targeting FBXW7 induces angiogenesis in BC tissues (Chiang et al., 2016). In addition to its involvement in regulating ubiquitin ligase (SCF) activity, tumor suppressor FBXW7 is essential for SCF complex activity, which controls the degradation of a variety of oncogenic proteins, such as HIF-1, Notch, cyclin E, and c-myc (Flügel et al., 2012). Thus, we conclude that miR-182 contributes to breast cancer invasion, angiogenesis, and metastasis based on the above studies.

### microR-210

miR-210, a hypoxia-inducing miRNA, has been primarily described as an oncomiR. Overexpression of miRNA-210 is a critical component of EC survival, angiogenesis, and differentiation in response to hypoxia (Fasanaro et al., 2008). In research conducted by Jung et al. (Jung et al., 2017), HIF-1 and miR-210 were overexpressed in exosomes produced from mouse BC cells during a hypoxic microenvironment. Exosomes carrying miR-210 were transfected into HUVEC cells, efficiently decreasing PTP1B and Ephrin-A3 expression and promoting angiogenesis by targeting VEGF signaling (Jung et al., 2017). MiR-210 expression was highly correlated with VEGF expression, hypoxia, and angiogenesis in breast cancer patients, suggesting that miR-210 may play a role in tumor angiogenesis. Although this association is significant, it is not adequate to evaluate whether miR-210 is a functional regulator of BC angiogenesis due to its substantial increase under hypoxic circumstances. An additional miR-210 target, the protein tyrosine phosphatase Ptp1b, has been discovered as a factor that promotes angiogenesis and suppresses cellular death in the setting of a mouse myocardial infarction (Hu et al., 2010). Earlier studies demonstrated that PTP1B might bind to and inhibit the activation of a VEGF receptor, VEGFR2, and inhibit the tyrosine phosphorylation of VEGFR2 in endothelial cells stabilizing cell-cell adhesions (Nakamura et al., 2008). Taken together, because of its ability to suppress Ptp1b and Efna3, miR-210 might facilitate angiogenesis.

### microR-467 

The miR-467 was found to be a specific inhibitor of TSP-1, which was reported to be elevated in the BC cells after glucose stimulation (Soheilifar et al., 2021). It was demonstrated that the miR-467 mimic increased the number of BC cells in the matrigel plugs in mice, indicating the proangiogenic activity of miR-467 *in vivo*. MiR-467, on the other hand, was unable to stimulate angiogenesis in the absence of TSP1Additionally, it was demonstrated that the amount of miR-467 in BC tumors increased, and a link between the expression miR-467 and tumor mass was shown in STZ-treated hyperglycemic mice. Similar results were found in the hyperglycemic Leprdb/db mice, with miRNA-467 hyperactivity leading to increased tumor growth and angiogenesis (Krukovets et al., 2015). Also, in the animal models, it was discovered that miRNA-467 blockers reduced tumor development and angiogenesis indicators (Krukovets et al., 2015). These findings show that hyperglycemia causes angiogenesis by increasing the expression of miR-467.

### microR26b and microR562

Both miR26b and miR562, tumor suppressor microRNAs, are found on human chromosomes 2q37.1 and 2q35, respectively. The expression of both NF-κB1 (p105) and NF-κB subunit RELA (p65) are directly repressed by miR26b and miR562, and this is linked with angiogenesis in breast cancer patients (Anbalagan et al., 2014). Many pathways, including the PI3K/AKT pathway, are associated with NF-κB signaling (Ghafouri-Fard et al., 2021a; Hussen et al., 2021b). In addition, the phosphatidyl inositol 3-kinase/Akt signaling is involved in the production of HIF-1 and VEGF, and it is essential for the development of blood vessels in the BC (Karar and Maity, 2011; Li et al., 2015). Because of this, miR-26b and miR-562 lead to BC angiogenesis through the activation of NF-κB, PI3K/AKT, HIF-1α, and VEGF pathways.

### microR-655/526b

The expression of miRNA-655/526b is considerably greater in human BC, and the higher expression of miR-655/526b is linked with a poorer prognosis (Gervin et al., 2020). *In vitro* research showed that COX2, an inflammatory enzyme elevated in BC, increases the expression of miR-655/526b (Majumder et al., 2015; Majumder et al., 2018). Furthermore, the researchers discovered that miR-655/526b transfection led to increases in the levels of angiogenic molecules like VEGFC, VEGF-D, COX2, and LYVE1 (Hunter et al., 2019). It was also shown that the expression of VEGFR1, which controls the growth of blood vessels, was increased in cell lines treated with both miRNAs. Additionally, HUVEC cells treated with medium containing miR-655/526b generated tubular structures (Hunter et al., 2019).

## Anti-angiogenic microRNA-Based Therapy

One of the most essential proposed strategies to combat and prevent cancer metastasis is to target angiogenesis pathways (Lee et al., 2015). Tumors start to produce a myriad of proangiogenic factors early during tumorigenesis to form their vasculature (Carmeliet and Jain, 2011), the anti-angiogenesis strategy, which was first suggested by Judah Folkman in 1971 now a day is considered an effective and promising antitumor strategy (Folkman, 1971), (Ebos and Kerbel, 2011). Therefore, targeting angiogenic miRNAs can be gained by either 1) miRNA-based therapeutics or 2) drugs and phytochemicals that already affect angiogenic miRNA. This category has additional advantages of being available and almost safe, and their toxicity and side effects are well studied (Varghese et al., 2020).

### microRNA-Based Therapeutics in Breast Cancer

miRNA-based therapeutics with antitumor and/or anti-angiogenic effects are achieved by either substituting or restoring tumor suppressor miRNAs activity or silencing overexpressed endogenous oncogenic miRNAs (Boca et al., 2020). In which mimic sequencing is used for restoring tumor suppressor miRNA (miRNA mimics) and exogenous delivery of antagonists (oligonucleotides that are chemically modified) for silencing endogenous oncogenic miRNAs (antagomir) (Caporali and Emanueli, 2011). Achieving efficient and targeted delivery of miRNAs mimics or antagomirs to targeted cancer tissues is of paramount importance. Some successful modalities have been researched extensively, yet a major obstacle still in progress is to be translated more successfully into clinical practice (Ganju et al., 2017). miRNA therapeutics can be delivered by either viral or non-viral vectors. Nano-technology delivery of miRNA therapeutics, a non-viral vector, sounds promising with less systemic toxicity and several types of nanocarriers being available in practice, each with a unique formulation, advantages, and disadvantages (Boca et al., 2020).

### Drugs and Phytochemicals Harboring Anti-angiogenic Activity in Breast Cancer

#### Melatonin

Melatonin is a flexible anti-cancer agent that has been studied extensively in various malignancies, including breast cancer. According to a recent study, melatonin has anticancer effects ranging from antiproliferative to increased apoptosis of breast cancer cells at physiological and pharmacological doses. Melatonin also has anti-metastatic properties and can reduce antitumor resistance and toxicity (Kong et al., 2020). Cheng et al. showed that melatonin reduces cellular viability and has an anti-angiogenic effect on HUVECs via the downregulation of the HIF1/ROS/VEGF axis, in which melatonin exerts these effects by directly inhibiting hypoxia-induced HIF1 and indirectly acting as a free radical scavenger, resulting in a reduction of ROS and VEGF crosstalk (Cheng et al., 2019).

Additionally, melatonin can also disrupt the development of vasculogenic mimicry (VM) via breast cancer cell lines in both normoxic and hypoxic conditions. VM mediates resistance toward anti-angiogenic drugs and has a significant role in breast cancer metastasis (Maroufi et al., 2020). Another study found that melatonin not only downregulates VEGF mRNA but also simultaneously downregulates ANG1 and ANG2 in breast cancer cells (González-González et al., 2018). miR-148a/152 overexpression is associated with marked inhibition of breast cancer cell proliferation and angiogenesis by targeting IGF-1R and IRS1 and consequently their downstream signaling pathway (Xu et al., 2013). Melatonin can also regulate the aforementioned angiogenic miRNAs, and it can upregulate expression of miR-152-3p, “a tumor suppressor found to be downregulated in breast cancer,” which in turn reduces the protein expression of IGF-insulin-like-like growth factor-1 receptor), HI, F-1,α, and VEGF (Marques et al., 2018). In another study, melatonin upregulated miR-148a-3 and reduced gene expression of IGF-1R and VEGF (Lacerda et al., 2019).

#### Metformin

Metformin, a widely used anti-diabetic, has been very extensively investigated in recent years for having several anti-cancer effects in breast cancer (DeCensi et al., 2014), (Goodwin et al., 2008), (Alimoradi et al., 2021). The most extensive ongoing clinical trial (NCT01101438) for using metformin in breast cancer will reveal metformin’s Role in breast cancer in detail. Metformin has several mechanisms for its beneficiary effect in breast cancer; among them, it affects miRNAs. Metformin exerted an anti-angiogenic effect in breast cancer models by inhibitiHER2-mediated VEGF upregulation and HIF-1α-mediated VEGF up-regulation, suggesting a novel mechanism of metformin targeting the HER2/HIF-1α/VEGF signaling axis (Wang et al., 2015).

Additionally, it was found out that metformin inhibited proliferation, tube formation, and migration of HUVECs by downregulation of miR-21 and TGF-β protein expression, consequently increasing PTEN and SMAD7 protein expression (Luo et al., 2017). Furthermore, Metformin was found to reduce breast cancer cell viability, upregulated miR-26a, and reduced expression of miR-26a targets PTEN and EHZ2 in several breast cancer cell lines (Cabello et al., 2016). Although, metformin might have an impact on metastasis, potentially via altering the levels of miR-21 in various cancer cell lines and breast cancer patients (Pulito et al., 2017). Metformin was also found to suppress miR-21 and miR-155 and up-regulate miR-200c in breast cancer cells, accordingly suppressing proliferation and metastasis of breast cancer (Alimoradi et al., 2021), (Sharma and Kumar, 2018), (Zhang et al., 2017).

These metformin effects showed synergism with everolimus, providing a potential role for metformin to be used in conjunction with breast cancer treatments.

#### Phytochemicals

Cardamonin suppressed miR-21 in HUVECs and accordingly suppressed VEGF-induced angiogenesis and cell migration (Jiang et al., 2015). Epigallocatechin-3-gallate (EGCG) from green tea and silibinin from milk thistle is widely consumed shown to have a powerful anti-angiogenic effect. Both synergistically, they were found to downregulate VEGF and miR-17–92 cluster while upregulated anti-angiogenic miR-19b in HUVECs (Mirzaaghaei et al., 2019). Interestingly EGCG was also found to inhibit tumor cell growth and angiogenesis via suppressing HIF-1α, NFκB a, and VEGF (Gu et al., 2013). Meanwhile, silibinin downregulated miR-21 and miR-155 in T47D breast cancer cells (Zadeh et al., 2015).

Furthermore, curcumin was found to suppress the proliferation and induce apoptosis of cancer cells (Ghaderi et al., 2021), (Sobhkhizi et al., 2020) as well as impinge MCF-7 cells by upregulating miR-15a and miR-16, which caused downregulation of Bcl-2 (Fix et al., 2010). Curcumin also upregulated miR-34a and miR-181b in breast cancer cell lines and inhibited invasion and metastasis of them (Guo et al., 2013), (Kronski et al., 2014), (Norouzi et al., 2018). In addition, curcumin efficiently targeted and elevated protein expression of miR-34a in MCF-10F and MDA-MB-231 cell lines and consequently affected regulatory genes of EMT and Rho-A and attenuated tumor cell migration and invasiveness (Gallardo et al., 2020). Beside of the above, several additional phytochemicals, such as resveratrol, luteolin, and betulinic acid, have been shown to influence angiogenic miRNAs (Varghese et al., 2020) (Table 3).

**TABLE 3:** Phytochemicals and their target miRNAs regulating tumor angiogenesis.

Compound	miRNA	Assessed cell line	Animal model	Target genes	Signaling Pathways	References
Melatonin	↑miR-152-3p	MDA-MB-468	Female BALC/c Nude mice	↓ angiogenesis by ↓IGF-1R	miR-152/IGF-IR, HIF-1α, VEGF pathway	Marques et al. (2018)
				↓HIF-1α, ↓VEGF		
	↑miR-148a-3	MDA-MB-231	Nude mice	↓ angiogenesis by ↓IGF-1R, ↓VEGF	IGF-1R/VEGF pathway	Lacerda et al. (2019)
				↓ migration, ↓ invasion of BC cells		
Metformin	↓miR-21	HUVEC	—	↓ migration, ↓ proliferation	TGF-β/PTEN/PI3k/AKT pathway	Luo et al. (2017)
				↓ angiogenesis, ↓ TGF-β		
				↑PTEN, ↑SMAD7		
	↑miR-26a	MDA-MB-468	—	↓ cell viability, ↓ Bcl-2	PTEN/AKT/PKB pathway	Cabello et al. (2016)
		MDA-MB-231		↓PTEN, ↓EHZ2		
		MCF-7				
	↓miR-181a	MCF-7	—	↓TGFβ and ↓ mamosphere-forming efficiency, ↓EMT	TGFβ-signaling pathway	Oliveras-Ferraros et al. (2011)
	↑miR-let-7a					
	↑miR-96					
	↓miR-21-5p	MCF-7	—	↑ AMPK, ↑CAB39L, ↑Sestrin-1 → ↓ mTOR synergistically with everolimus → ↓ cell invasion and growth	AMPK/mTOR pathway	Pulito et al. (2017)
		BT-549				
		BT-474, SUM159PT				
	↓miR-21	MDA-MB-23	—	↓ ROS → ↑ SOD	ROS-independent pathway	Sharma and Kumar (2018)
	↓miR-155	MCF-7		↓ MMP-2, ↓MMP-9, ↓ Bcl-2		
				↑ apoptosis and ↓ proliferation		
	↑miR-200c	MDA-MB-231	SCID mice	↓ growth, migration and invasion, ↑ apoptosis	metformin/c-Myc/miR-200c/AKT2/Bcl-2 pathway	Zhang et al. (2017)
		MCF-7, BT549, T-47-D		↓AKT2, ↓ c-Myc, ↓Bcl-2		
Cardamonin	↓miR-21	HUVEC	—	↓VEGF mediated angiogenesis	miR-21/VEGF signaling	Jiang et al. (2015)
				↓ proliferation and migration of endothelial cells		
(Silibilin + EGCG)	↓ miR-17	HUVEC A549	—	Synergistically ↓VEGF and ↓VEGFR2	VEGF/VEGFR2 pathway	Mirzaaghaei et al. (2019)
	↓ miR-18a					
	↓ miR-20a					
	↑miR-19b					
	↑miR-92a					
Curcumin	↑miR-15a	MCF-7	—	↓Bcl-2	miR-15a/16-Bcl-2 apoptotic pathway	Yang et al. (2010)
	↑miR-16					
	↑miR-34a	MDA-MB-231	—	↓ proliferation, survival, invasion and ↑apoptosis	Bcl-2 mediated apoptotic pathway	Guo et al. (2013)
		MDA-MB-435		↓ Bcl-2, ↓Bmi-1		
	↑miR-181b	MDA‐MB‐231	—	↓proliferation, ↑apoptosis by ↓ Bcl-2, ↓survivin, ↓MMP-1, ↓MMP-3	Bcl-2 mediated apoptotic pathway	Kronski et al. (2014)
				Anti-metastatic effect by ↓CXCL1, ↓CXCL 2		
	↑miR-34a	MCF-10F	—	↓ cell viability, ↓ cell migration and invasive ness	Rho -signaling pathway	Gallardo et al. (2020)
		MDA-MB-231		↓EMT (Axl, Slug, CD24)		
				↓Rho-A		
	↓miR-21	MCF-7	—	↑ caspase 3/9→ ↑ apoptosis	miR-21/PTEN/Akt pathway	Wang et al. (2017)
				↑ PTEN, ↓ p-AKT		
Hesperidin and luteolin	↓miR-21	MCF-7	—	↓ cell viability, ↑ apoptosis, ↓Bcl-2	Bcl2/Bax apoptotic pathway	Magura et al. (2020)
	↑ miR-16			↑ Bax		
	↑ miR-34a					
Quercetin	↓miR-21	MCF-7	—	↑Maspin, ↑PTEN, ↓ cell viability	PTEN/maspin pathway	Panahi, (2018)
betulinic acid	↓miR-27a	MDA-MB-231, BT-549	—	↑ ZBTB10, ↓Sp1, Sp3, Sp4	miR-27a/ZBTB10/Sp-axis	Talcott et al. (2008)
				↑ Myt-1		
	↓miR-27a	MDA-MB-231	Female athymic Nude mice	↓ angiogenesis by ↓Sp1, Sp3 and Sp4, ↑ ZBTB10	miR-27a/ZBTB10/Sp-axis	Mertens-Talcott et al. (2013)
				↑ cell cycle arrest in G2/M phase		
				↑ Myt-1, ↓VEGFR, and ↓survivin in mice		
				↓ hβ2G in lung of mice		
Glabridin	↑miR-148a	MDA-MB-231	—	↓ angiogenesis by ↓Wnt/β-catenin pathway and ↓ VEGF secretion	miR-148a/Wnt/β-catenin signaling	Alhasan, (2019)
		Hs-578T				
Pomegranate	↓ miR-27a	BT474	Female athymic BALB/c Nude mice	↓ Sp1, Sp3 and Sp4 → ↑ ZBTB10	miR-27a/ZBTB10/Sp-axis	Banerjee et al. (2012)
	↓miR-155	MDA-MB-231		↓ cyclin D1, ↓Bcl-2, ↓surviving	miR-155/SHIP1/PIP3/AKT/NF-kB- axis	
				↓ VEGF and VEGF1-R, ↓ NF-_k_B		
				↑ SHIP-1→↓ pPI3K and ↓ pAKT		
				↓ NF-_k_B		
Mango	↑miR-126	BT474	Female athymic BALB/c Nude mice	↓PI3k/AKT pathway, ↓HIF-1α, ↓VEGF, ↓ NF-_k_B, ↓ mTOR	miRNA-126/PI3K/AKT -axis	Banerjee et al. (2015)

## Conclusions and Future Perspectives

MicroRNAs regulate tumor angiogenesis, a key component in cancer growth and metastasis. Identification of novel molecular features of angiogenesis regulation, and a greater understanding of cancer progression strategies, will allow the development of new therapeutic options. Numerous genes which involved in angiogenesis are regulated by miRNAs, therefore identifying miRNA-target interaction networks might be useful in describing anti-angiogenic therapy and novel diagnostic biomarkers in BC. Angio-regulatory miRNAs may be used to produce a new generation of medicines such as nano-based therapeutics. Additionally, phytochemical medicines might modulate the expression of angio-regulatory miRs, which in turn could enhance survival in BC patients. Furthermore, we revealed the inhibiting and stimulating pathways of angio-regulatory miRNAs in cancer-related angiogenesis process, which may be useful in the developing anti-angiogenic methods in cancer therapy.

## References

[B1] Aguilar-Cazares D. Chavez-Dominguez R. Carlos-Reyes A. Lopez-Camarillo C. Hernadez de la Cruz O. N. Lopez-Gonzalez J. S. (2019). Contribution of Angiogenesis to Inflammation and Cancer. Front. Oncol. 9, 1399. 10.3389/fonc.2019.01399 31921656 PMC6920210

[B2] Alhasan L. (2019). MiR-126 Modulates Angiogenesis in Breast Cancer by Targeting VEGF-A -mRNA. Asian Pac. J. Cancer Prev. 20 (1), 193–197. 10.31557/apjcp.2019.20.1.193 30678431 PMC6485552

[B3] Alimoradi N. Firouzabadi N. Fatehi R. (2021). How Metformin Affects Various Malignancies by Means of microRNAs: a Brief Review. Cancer Cel Int 21 (1), 207. 10.1186/s12935-021-01921-z PMC8045276 33849540

[B4] Alles J. Fehlmann T. Fischer U. Backes C. Galata V. Minet M. (2019). An Estimate of the Total Number of True Human miRNAs. Nucleic Acids Res. 47 (7), 3353–3364. 10.1093/nar/gkz097 30820533 PMC6468295

[B5] Anbalagan D. Yap G. Yuan Y. Pandey V. K. Lau W. H. Arora S. (2014). Annexin-A1 Regulates MicroRNA-26b* and MicroRNA-562 to Directly Target NF-Κb and Angiogenesis in Breast Cancer Cells. PLoS One 9 (12), e114507. 10.1371/journal.pone.0114507 25536365 PMC4275173

[B6] Babashah S. Soleimani M. (2011). The Oncogenic and Tumour Suppressive Roles of microRNAs in Cancer and Apoptosis. Eur. J. Cancer 47 (8), 1127–1137. 10.1016/j.ejca.2011.02.008 21402473

[B7] Baeriswyl V. Christofori G. (2009). The Angiogenic Switch in Carcinogenesis. Semin. Cancer Biol. 19 (5), 329–337. 10.1016/j.semcancer.2009.05.003 19482086

[B8] Bakr N. M. Mahmoud M. S. Nabil R. Boushnak H. Swellam M. (2021). Impact of Circulating miRNA-373 on Breast Cancer Diagnosis through Targeting VEGF and Cyclin D1 Genes. J. Genet. Eng. Biotechnol. 19 (1), 84. 10.1186/s43141-021-00174-7 34089425 PMC8179880

[B9] Banerjee N. Kim H. Krenek K. Talcott S. T. Mertens-Talcott S. U. (2015). Mango Polyphenolics Suppressed Tumor Growth in Breast Cancer Xenografts in Mice: Role of the PI3K/AKT Pathway and Associated microRNAs. Nutr. Res. 35 (8), 744–751. 10.1016/j.nutres.2015.06.002 26194618

[B10] Banerjee N. Talcott S. Safe S. Mertens-Talcott S. U. (2012). Cytotoxicity of Pomegranate Polyphenolics in Breast Cancer Cells *In Vitro* and Vivo: Potential Role of miRNA-27a and miRNA-155 in Cell Survival and Inflammation. Breast Cancer Res. Treat. 136 (1), 21–34. 10.1007/s10549-012-2224-0 22941571 PMC3488590

[B11] Bartel D. P. (2004). MicroRNAs. Cell 116 (2), 281–297. 10.1016/s0092-8674(04)00045-5 14744438

[B12] Bentley K. Chakravartula S. (2017). The Temporal Basis of Angiogenesis. Phil. Trans. R. Soc. B 372 (1720), 20150522. 10.1098/rstb.2015.0522 28348255 PMC5379027

[B13] Bertoli G. Cava C. Castiglioni I. (2015). MicroRNAs: New Biomarkers for Diagnosis, Prognosis, Therapy Prediction and Therapeutic Tools for Breast Cancer. Theranostics 5 (10), 1122–1143. 10.7150/thno.11543 26199650 PMC4508501

[B14] Bitaraf A. Babashah S. Garshasbi M. (2020). Aberrant Expression of a Five-microRNA Signature in Breast Carcinoma as a Promising Biomarker for Diagnosis. J. Clin. Lab. Anal. 34 (2), e23063. 10.1002/jcla.23063 31595567 PMC7031575

[B15] Boca S. Gulei D. Zimta A.-A. Onaciu A. Magdo L. Tigu A. B. (2020). Nanoscale Delivery Systems for microRNAs in Cancer Therapy. Cell. Mol. Life Sci. 77 (6), 1059–1086. 10.1007/s00018-019-03317-9 31637450 PMC11105078

[B16] Bohnsack M. T. Czaplinski K. Gorlich D. (2004). Exportin 5 Is a RanGTP-dependent dsRNA-Binding Protein that Mediates Nuclear export of Pre-miRNAs. RNA 10 (2), 185–191. 10.1261/rna.5167604 14730017 PMC1370530

[B17] Broughton J. P. Lovci M. T. Huang J. L. Yeo G. W. Pasquinelli A. E. (2016). Pairing beyond the Seed Supports MicroRNA Targeting Specificity. Mol. Cel 64 (2), 320–333. 10.1016/j.molcel.2016.09.004 PMC5074850 27720646

[B18] Bu P. Luo C. He Q. Yang P. Li X. Xu D. (2017). MicroRNA-9 Inhibits the Proliferation and Migration of Malignant Melanoma Cells via Targeting Sirituin 1. Exp. Ther. Med. 14 (2), 931–938. 10.3892/etm.2017.4595 28810544 PMC5526066

[B19] Cabello P. Pineda B. Tormo E. Lluch A. Eroles P. (2016). The Antitumor Effect of Metformin is Mediated by miR-26a in Breast Cancer. Int. J. Mol. Sci. 17 (8), 1298. 10.3390/ijms17081298 PMC5000695 27517917

[B20] Caporali A. Emanueli C. (2011). MicroRNA Regulation in Angiogenesis. Vasc. Pharmacol. 55 (4), 79–86. 10.1016/j.vph.2011.06.006 21777698

[B21] Carmeliet P. Jain R. K. (2000). Angiogenesis in Cancer and Other Diseases. Nature 407 (6801), 249–257. 10.1038/35025220 11001068

[B22] Carmeliet P. Jain R. K. (2011). Molecular Mechanisms and Clinical Applications of Angiogenesis. Nature 473 (7347), 298–307. 10.1038/nature10144 21593862 PMC4049445

[B23] Carthew R. W. Sontheimer E. J. (2009). Origins and Mechanisms of miRNAs and siRNAs. Cell 136 (4), 642–655. 10.1016/j.cell.2009.01.035 19239886 PMC2675692

[B24] Cascio S. D'Andrea A. Ferla R. Surmacz E. Gulotta E. Amodeo V. (2010). miR-20b Modulates VEGF Expression by Targeting HIF-1 Alpha and STAT3 in MCF-7 Breast Cancer Cells. J. Cel Physiol 224 (1), 242–249. 10.1002/jcp.22126 20232316

[B25] Cha S.-T. Chen P.-S. Johansson G. Chu C.-Y. Wang M.-Y. Jeng Y.-M. (2010). MicroRNA-519c Suppresses Hypoxia-Inducible Factor-1α Expression and Tumor Angiogenesis. Cancer Res. 70 (7), 2675–2685. 10.1158/0008-5472.can-09-2448 20233879

[B26] Chaffer C. L. Weinberg R. A. (2011). A Perspective on Cancer Cell Metastasis. Science 331 (6024), 1559–1564. 10.1126/science.1203543 21436443

[B27] Chang S. Wang R. H. Wang R.-H. Akagi K. Kim K.-A. Martin B. K. (2011). Tumor Suppressor BRCA1 Epigenetically Controls Oncogenic microRNA-155. Nat. Med. 17 (10), 1275–1282. 10.1038/nm.2459 21946536 PMC3501198

[B28] Chen H.-X. Xu X.-X. Tan B.-Z. Zhang Z. Zhou X.-D. (2017). MicroRNA-29b Inhibits Angiogenesis by Targeting VEGFA through the MAPK/ERK and PI3K/Akt Signaling Pathways in Endometrial Carcinoma. Cell Physiol Biochem 41 (3), 933–946. 10.1159/000460510 28222438

[B29] Chen J. Wang B.-C. Tang J.-H. (2012). Clinical Significance of MicoRNA-155 Expression in Human Breast Cancer. J. Surg. Oncol. 106 (3), 260–266. 10.1002/jso.22153 22105810

[B30] Chen S. Xue Y. Wu X. Le C. Bhutkar A. Bell E. L. (2014). Global microRNA Depletion Suppresses Tumor Angiogenesis. Genes Dev. 28 (10), 1054–1067. 10.1101/gad.239681.114 24788094 PMC4035535

[B31] Cheng J. Yang H. L. Gu C. J. Liu Y. K. Shao J. Zhu R. (2019). Melatonin Restricts the Viability and Angiogenesis of Vascular Endothelial Cells by Suppressing HIF-1α/ROS/VEGF. Int. J. Mol. Med. 43 (2), 945–955. 10.3892/ijmm.2018.4021 30569127 PMC6317691

[B32] Chiang C. H. Chu P. Y. Hou M. F. Hung W. C. (2016). MiR-182 Promotes Proliferation and Invasion and Elevates the HIF-1α-VEGF-A axis in Breast Cancer Cells by Targeting FBXW7. Am. J. Cancer Res. 6 (8), 1785–1798. 27648365 PMC5004079

[B33] Choi Y.-C. Yoon S. Jeong Y. Yoon J. Baek K. (2011). Regulation of Vascular Endothelial Growth Factor Signaling by miR-200b. Mol. Cell 32 (1), 77–82. 10.1007/s10059-011-1042-2 PMC3887663 21544626

[B34] Danza K. Summa S. D. Pinto R. Pilato B. Palumbo O. Merla G. (2015). MiR-578 and miR-573 as Potential Players in BRCA-Related Breast Cancer Angiogenesis. Oncotarget 6 (1), 471–483. 10.18632/oncotarget.2509 25333258 PMC4381608

[B35] Dastmalchi N. Safaralizadeh R. Latifi-Navid S. Banan Khojasteh S. M. Mahmud Hussen B. Teimourian S. (2021). An Updated Review of the Role of lncRNAs and Their Contribution in Various Molecular Subtypes of Breast Cancer. Expert Rev. Mol. Diagn. 10.1080/14737159.2021.1962707 34334086

[B36] Dayan F. Mazure N. M. Brahimi-Horn M. C. Pouysségur J. (2008). A Dialogue between the Hypoxia-Inducible Factor and the Tumor Microenvironment. Cancer Microenvironment 1 (1), 53–68. 10.1007/s12307-008-0006-3 19308685 PMC2654353

[B37] de Sales A. C. V. da Silva I. Leite M. C. B. de Lima Coutinho L. de Albuquerque Cavalcante Reis R. B. Martins D. B. G. (2021). miRNA-195 Expression in the Tumor Tissues of Female Brazilian Breast Cancer Patients with Operable Disease. Clinics (Sao Paulo). 76, e2142. 10.6061/clinics/2021/e2142 33503182 PMC7798133

[B38] DeCensi A. Puntoni M. Gandini S. Guerrieri-Gonzaga A. Johansson H. A. Cazzaniga M. (2014). Differential Effects of Metformin on Breast Cancer Proliferation According to Markers of Insulin Resistance and Tumor Subtype in a Randomized Presurgical Trial. Breast Cancer Res. Treat. 148 (1), 81–90. 10.1007/s10549-014-3141-1 25253174 PMC4196136

[B39] Dong L. Zhou D. Xin C. Liu B. Sun P. (2020). MicroRNA-139 Suppresses the Tumorigenicity of Triple Negative Breast Cancer Cells by Targeting SOX8. Cmar 12, 9417–9428. 10.2147/cmar.s268378 PMC7535124 33061629

[B40] Ebos J. M. L. Kerbel R. S. (2011). Antiangiogenic Therapy: Impact on Invasion, Disease Progression, and Metastasis. Nat. Rev. Clin. Oncol. 8 (4), 210–221. 10.1038/nrclinonc.2011.21 21364524 PMC4540336

[B41] Fabbri E. Montagner G. Bianchi N. Finotti A. Borgatti M. Lampronti I. (2016). MicroRNA miR-93-5p Regulates Expression of IL-8 and VEGF in Neuroblastoma SK-N-AS Cells. Oncol. Rep. 35 (5), 2866–2872. 10.3892/or.2016.4676 26986724

[B42] Fan X. Fang X. Liu G. Xiong Q. Li Z. Zhou W. (2019). MicroRNA-204 Inhibits the Proliferation and Metastasis of Breast Cancer Cells by Targeting PI3K/AKT Pathway. J. Buon 24 (3), 1054–1059. 31424660

[B43] Fang J.-H. Zhou H.-C. Zeng C. Yang J. Liu Y. Huang X. (2011). MicroRNA-29b Suppresses Tumor Angiogenesis, Invasion, and Metastasis by Regulating Matrix Metalloproteinase 2 Expression. Hepatology 54 (5), 1729–1740. 10.1002/hep.24577 21793034

[B44] Fang L. Du W. W. Yang W. Rutnam Z. J. Peng C. Li H. (2012). MiR-93 Enhances Angiogenesis and Metastasis by Targeting LATS2. Cell Cycle 11 (23), 4352–4365. 10.4161/cc.22670 23111389 PMC3552918

[B45] Fasanaro P. D'Alessandra Y. Di Stefano V. Melchionna R. Romani S. Pompilio G. (2008). MicroRNA-210 Modulates Endothelial Cell Response to Hypoxia and Inhibits the Receptor Tyrosine Kinase Ligand Ephrin-A3. J. Biol. Chem. 283 (23), 15878–15883. 10.1074/jbc.m800731200 18417479 PMC3259646

[B46] Fish J. E. Santoro M. M. Morton S. U. Yu S. Yeh R.-F. Wythe J. D. (2008). miR-126 Regulates Angiogenic Signaling and Vascular Integrity. Dev. Cel 15 (2), 272–284. 10.1016/j.devcel.2008.07.008 PMC2604134 18694566

[B47] Fix L. N. Shah M. Efferth T. Farwell M. A. Zhang B. (2010). MicroRNA Expression Profile of MCF-7 Human Breast Cancer Cells and the Effect of green tea Polyphenon-60. Cancer Genomics Proteomics 7 (5), 261–277. 20952761

[B48] Flores-Pérez A. Marchat L. A. Rodríguez-Cuevas S. Bautista-Piña V. Hidalgo-Miranda A. Ocampo E. A. (2016). Dual Targeting of ANGPT1 and TGFBR2 Genes by miR-204 Controls Angiogenesis in Breast Cancer. Sci. Rep. 6, 34504. 10.1038/srep34504 27703260 PMC5050489

[B49] Flügel D. Görlach A. Kietzmann T. (2012). GSK-3β Regulates Cell Growth, Migration, and Angiogenesis via Fbw7 and USP28-dependent Degradation of HIF-1α. Blood 119 (5), 1292–1301. 10.1182/blood-2011-08-375014 22144179 PMC3352078

[B50] Foekens J. A. Sieuwerts A. M. Smid M. Look M. P. de Weerd V. Boersma A. W. M. (2008). Four miRNAs Associated with Aggressiveness of Lymph Node-Negative, Estrogen Receptor-Positive Human Breast Cancer. Proc. Natl. Acad. Sci. 105 (35), 13021–13026. 10.1073/pnas.0803304105 18755890 PMC2529088

[B51] Folkman J. (1971). Tumor Angiogenesis: Therapeutic Implications. N. Engl. J. Med. 285 (21), 1182–1186. 10.1056/NEJM197111182852108 4938153

[B52] Fong G. H. Zhang L. Bryce D. M. Peng J. (1999). Increased Hemangioblast Commitment, Not Vascular Disorganization, Is the Primary Defect in Flt-1 Knock-Out Mice. Development 126 (13), 3015–3025. 10.1242/dev.126.13.3015 10357944

[B53] Gallardo M. Kemmerling U. Aguayo F. Bleak T. C. Muñoz J. P. Calaf G. M. (2020). Curcumin Rescues Breast Cells from Epithelial-mesenchymal T-ransition and I-nvasion I-nduced by anti-miR-34a. Int. J. Oncol. 56 (2), 480–493. 10.3892/ijo.2019.4939 31894298 PMC6959390

[B54] Ganju A. Khan S. Hafeez B. B. Behrman S. W. Yallapu M. M. Chauhan S. C. (2017). miRNA Nanotherapeutics for Cancer. Drug Discov. Today 22 (2), 424–432. 10.1016/j.drudis.2016.10.014 27815139 PMC5309208

[B55] Gervin E. Shin B. Opperman R. Cullen M. Feser R. Maiti S. (2020). Chemically Induced Hypoxia Enhances miRNA Functions in Breast Cancer. Cancers 12 (8), 2008. 10.3390/cancers12082008 PMC7465874 32707933

[B56] Ghaderi S. Babaei E. Hussen B. M. Mahdavi M. Azeez H. J. (2021). Gemini Curcumin Suppresses Proliferation of Ovarian Cancer OVCAR-3 Cells via Induction of Apoptosis. Acamc 21 (6), 775–781. 10.2174/1871520620666200807223340 32767955

[B57] Ghafouri-Fard S. Abak A. Fattahi F. Hussen B. M. Bahroudi Z. Shoorei H. (2021). The Interaction between miRNAs/lncRNAs and Nuclear Factor-Κb (NF-Κb) in Human Disorders. Biomed. Pharmacother. 138, 111519. 10.1016/j.biopha.2021.111519 33756159

[B58] Ghafouri-Fard S. Glassy M. C. Abak A. Hussen B. M. Niazi V. Taheri M. (2021). The Interaction between miRNAs/lncRNAs and Notch Pathway in Human Disorders. Biomed. Pharmacother. 138, 111496. 10.1016/j.biopha.2021.111496 33743335

[B59] González-González A. González A. Alonso-González C. Menéndez-Menéndez J. Martínez-Campa C. Cos S. (2018). Complementary Actions of Melatonin on Angiogenic Factors, the angiopoietin/Tie2 axis and VEGF, in Co-cultures of H-uman E-ndothelial and B-reast C-ancer C-ells. Oncol. Rep. 39 (1), 433–441. 10.3892/or.2017.6070 29115538

[B60] Good D. J. Polverini P. J. Rastinejad F. Le Beau M. M. Lemons R. S. Frazier W. A. (1990). A Tumor Suppressor-dependent Inhibitor of Angiogenesis Is Immunologically and Functionally Indistinguishable from a Fragment of Thrombospondin. Proc. Natl. Acad. Sci. 87 (17), 6624–6628. 10.1073/pnas.87.17.6624 1697685 PMC54589

[B61] Goodwin P. J. Pritchard K. I. Ennis M. Clemons M. Graham M. Fantus I. G. (2008). Insulin-lowering Effects of Metformin in Women with Early Breast Cancer. Clin. Breast Cancer 8 (6), 501–505. 10.3816/cbc.2008.n.060 19073504

[B62] Goradel N. H. Mohammadi N. Haghi‐Aminjan H. Farhood B. Negahdari B. Sahebkar A. (2019). Regulation of Tumor Angiogenesis by microRNAs: State of the Art. J. Cel Physiol 234 (2), 1099–1110. 10.1002/jcp.27051 30070704

[B63] Gregory R. I. Chendrimada T. P. Cooch N. Shiekhattar R. (2005). Human RISC Couples microRNA Biogenesis and Posttranscriptional Gene Silencing. Cell 123 (4), 631–640. 10.1016/j.cell.2005.10.022 16271387

[B64] Gu J.-W. Makey K. L. Tucker K. B. Chinchar E. Mao X. Pei I. (2013). EGCG, a Major green tea Catechin Suppresses Breast Tumor Angiogenesis and Growth via Inhibiting the Activation of HIF-1α and NFκB, and VEGF Expression. Vasc. Cel 5 (1), 9. 10.1186/2045-824x-5-9 PMC3649947 23638734

[B65] Guo J. Li W. Shi H. Xie X. Li L. Tang H. (2013). Synergistic Effects of Curcumin with Emodin against the Proliferation and Invasion of Breast Cancer Cells through Upregulation of miR-34a. Mol. Cel Biochem 382 (1-2), 103–111. 10.1007/s11010-013-1723-6 23771315

[B66] Guttilla I. K. White B. A. (2009). Coordinate Regulation of FOXO1 by miR-27a, miR-96, and miR-182 in Breast Cancer Cells. J. Biol. Chem. 284 (35), 23204–23216. 10.1074/jbc.m109.031427 19574223 PMC2749094

[B67] Hao Y. Yang J. Yin S. Zhang H. Fan Y. Sun C. (2014). The Synergistic Regulation of VEGF-Mediated Angiogenesis through miR-190 and Target Genes. RNA 20 (8), 1328–1336. 10.1261/rna.044651.114 24962367 PMC4105756

[B68] Harris T. A. Yamakuchi M. Ferlito M. Mendell J. T. Lowenstein C. J. (2008). MicroRNA-126 Regulates Endothelial Expression of Vascular Cell Adhesion Molecule 1. Proc. Natl. Acad. Sci. 105 (5), 1516–1521. 10.1073/pnas.0707493105 18227515 PMC2234176

[B69] Hashimoto T. Shibasaki F. (2015). Hypoxia-Inducible Factor as an Angiogenic Master Switch. Front. Pediatr. 3 (33), 33. 10.3389/fped.2015.00033 25964891 PMC4408850

[B70] Hassel D. Cheng P. White M. P. Ivey K. N. Kroll J. Augustin H. G. (2012). MicroRNA-10 Regulates the Angiogenic Behavior of Zebrafish and Human Endothelial Cells by Promoting Vascular Endothelial Growth Factor Signaling. Circ. Res. 111 (11), 1421–1433. 10.1161/circresaha.112.279711 22955733 PMC3525481

[B71] He T. Qi F. Jia L. Wang S. Song N. Guo L. (2014). MicroRNA-542-3p Inhibits Tumour Angiogenesis by Targeting Angiopoietin-2. J. Pathol. 232 (5), 499–508. 10.1002/path.4324 24403060

[B72] He T. Qi F. Jia L. Wang S. Wang C. Song N. (2015). Tumor Cell-Secreted Angiogenin Induces Angiogenic Activity of Endothelial Cells by Suppressing miR-542-3p. Cancer Lett. 368 (1), 115–125. 10.1016/j.canlet.2015.07.036 26272182

[B73] Holmes D. I. Zachary I. (2005). The Vascular Endothelial Growth Factor (VEGF) Family: Angiogenic Factors in Health and Disease. Genome Biol. 6 (2), 209. 10.1186/gb-2005-6-2-209 15693956 PMC551528

[B74] Hong B. S. Ryu H. S. Kim N. Kim J. Lee E. Moon H. (2019). Tumor Suppressor miRNA-204-5p Regulates Growth, Metastasis, and Immune Microenvironment Remodeling in Breast Cancer. Cancer Res. 79 (7), 1520–1534. 10.1158/0008-5472.CAN-18-0891 30737233

[B75] Hong Z. Hong C. Ma B. Wang Q. Zhang X. Li L. (2019). MicroRNA-126-3p I-nhibits the P-roliferation, M-igration, I-nvasion, and A-ngiogenesis of T-riple-negative B-reast C-ancer C-ells by T-argeting RGS3. Oncol. Rep. 42 (4), 1569–1579. 10.3892/or.2019.7251 31364749

[B76] Hu S. Huang M. Li Z. Jia F. Ghosh Z. Lijkwan M. A. (2010). MicroRNA-210 as a Novel Therapy for Treatment of Ischemic Heart Disease. Circulation 122 (11 Suppl. l), S124–S131. 10.1161/CIRCULATIONAHA.109.928424 20837903 PMC2952325

[B77] Hunter S. Nault B. Ugwuagbo K. C. Maiti S. Majumder M. (2019). Mir526b and Mir655 Promote Tumour Associated Angiogenesis and Lymphangiogenesis in Breast Cancer. Cancers (Basel) 11 (7), 938. 10.3390/cancers11070938 PMC6678879 31277414

[B78] Hussen B. M. Azimi T. Hidayat H. J. Taheri M. Ghafouri-Fard S. (2021). NF-KappaB Interacting LncRNA: Review of its Roles in Neoplastic and Non-neoplastic Conditions. Biomed. Pharmacother. 139, 111604. 10.1016/j.biopha.2021.111604 33895520

[B79] Hussen B. M. Hidayat H. J. Salihi A. Sabir D. K. Taheri M. Ghafouri-Fard S. (2021). MicroRNA: A Signature for Cancer Progression. Biomed. Pharmacother. 138, 111528. 10.1016/j.biopha.2021.111528 33770669

[B80] Isanejad A. Alizadeh A. M. Amani Shalamzari S. Khodayari H. Khodayari S. Khori V. (2016). MicroRNA-206, Let-7a and microRNA-21 Pathways Involved in the Anti-angiogenesis Effects of the Interval Exercise Training and Hormone Therapy in Breast Cancer. Life Sci. 151, 30–40. 10.1016/j.lfs.2016.02.090 26924493

[B81] Jiang F. S. Tian S. S. Lu J. J. Ding X. H. Qian C. D. Ding B. (2015). Cardamonin Regulates miR-21 Expression and Suppresses Angiogenesis Induced by Vascular Endothelial Growth Factor. Biomed. Res. Int. 2015, 501581. 10.1155/2015/501581 26266258 PMC4523674

[B82] Jin X. Dai L. Ma Y. Wang J. Liu Z. (2020). Implications of HIF-1α in the Tumorigenesis and Progression of Pancreatic Cancer. Cancer Cel Int 20 (1), 273. 10.1186/s12935-020-01370-0 PMC7313137 32587480

[B83] Jung K. O. Youn H. Lee C.-H. Kang K. W. Chung J.-K. (2017). Visualization of Exosome-Mediated miR-210 Transfer from Hypoxic Tumor Cells. Oncotarget 8 (6), 9899–9910. 10.18632/oncotarget.14247 28038441 PMC5354779

[B84] Karar J. Maity A. (2011). PI3K/AKT/mTOR Pathway in Angiogenesis. Front. Mol. Neurosci. 4 (51), 51. 10.3389/fnmol.2011.00051 22144946 PMC3228996

[B85] Ke K. Lou T. (2017). MicroRNA-10a Suppresses Breast Cancer Progression via PI3K/Akt/mTOR Pathway. Oncol. Lett. 14 (5), 5994–6000. 10.3892/ol.2017.6930 29113237 PMC5661611

[B86] Kim B. G. Gao M.-Q. Kang S. Choi Y. P. Lee J. H. Kim J. E. (2017). Mechanical Compression Induces VEGFA Overexpression in Breast Cancer via DNMT3A-dependent miR-9 Downregulation. Cell Death Dis 8 (3), e2646. 10.1038/cddis.2017.73 28252641 PMC5386566

[B87] Kim H. Ko Y. Park H. Zhang H. Jeong Y. Kim Y. (2019). MicroRNA-148a/b-3p Regulates Angiogenesis by Targeting Neuropilin-1 in Endothelial Cells. Exp. Mol. Med. 51 (11), 1–11. 10.1038/s12276-019-0344-x PMC6853980 31723119

[B88] Kong W. He L. Richards E. J. Challa S. Xu C.-X. Permuth-Wey J. (2014). Upregulation of miRNA-155 Promotes Tumour Angiogenesis by Targeting VHL and Is Associated with Poor Prognosis and Triple-Negative Breast Cancer. Oncogene 33 (6), 679–689. 10.1038/onc.2012.636 23353819 PMC3925335

[B89] Kong X. Gao R. Wang Z. Wang X. Fang Y. Gao J. (2020). Melatonin: A Potential Therapeutic Option for Breast Cancer. Trends Endocrinol. Metab. 31 (11), 859–871. 10.1016/j.tem.2020.08.001 32893084

[B90] Kronski E. Fiori M. E. Barbieri O. Astigiano S. Mirisola V. Killian P. H. (2014). miR181b Is Induced by the Chemopreventive Polyphenol Curcumin and Inhibits Breast Cancer Metastasis via Down-Regulation of the Inflammatory Cytokines CXCL1 and -2. Mol. Oncol. 8 (3), 581–595. 10.1016/j.molonc.2014.01.005 24484937 PMC5528633

[B91] Krukovets I. Legerski M. Sul P. Stenina-Adognravi O. (2015). Inhibition of Hyperglycemia-Induced Angiogenesis and Breast Cancer Tumor Growth by Systemic Injection of microRNA-467 Antagonist. FASEB J. 29 (9), 3726–3736. 10.1096/fj.14-267799 26018675 PMC4550367

[B92] Kuehbacher A. Urbich C. Dimmeler S. (2008). Targeting microRNA Expression to Regulate Angiogenesis. Trends Pharmacol. Sci. 29 (1), 12–15. 10.1016/j.tips.2007.10.014 18068232

[B93] Kuehbacher A. Urbich C. Zeiher A. M. Dimmeler S. (2007). Role of Dicer and Drosha for Endothelial microRNA Expression and Angiogenesis. Circ. Res. 101 (1), 59–68. 10.1161/circresaha.107.153916 17540974

[B94] Lacerda J. Z. Ferreira L. C. Lopes B. C. Aristizábal-Pachón A. F. Bajgelman M. C. Borin T. F. (2019). Therapeutic Potential of Melatonin in the Regulation of MiR-148a-3p and Angiogenic Factors in Breast Cancer. Mirna 8 (3), 237–247. 10.2174/2211536608666190219095426 30806335

[B95] Lai Y. Quan J. Lin C. Li H. Hu J. Chen P. (2018). miR-199b-5p Serves as a Tumor Suppressor in Renal Cell Carcinoma. Exp. Ther. Med. 16 (1), 436–444. 10.3892/etm.2018.6151 29896270 PMC5995031

[B96] Lee R. C. Feinbaum R. L. Ambros V. (1993). The *C. elegans* Heterochronic Gene Lin-4 Encodes Small RNAs with Antisense Complementarity to Lin-14. Cell 75 (5), 843–854. 10.1016/0092-8674(93)90529-y 8252621

[B97] Lee S. H. Jeong D. Han Y.-S. Baek M. J. (2015). Pivotal Role of Vascular Endothelial Growth Factor Pathway in Tumor Angiogenesis. Ann. Surg. Treat. Res. 89 (1), 1–8. 10.4174/astr.2015.89.1.1 26131438 PMC4481026

[B98] Lei R. Tang J. Zhuang X. Deng R. Li G. Yu J. (2014). Suppression of MIM by microRNA-182 Activates RhoA and Promotes Breast Cancer Metastasis. Oncogene 33 (10), 1287–1296. 10.1038/onc.2013.65 23474751

[B99] Leidner R. S. Li L. Thompson C. L. (2013). Dampening Enthusiasm for Circulating microRNA in Breast Cancer. PLoS One 8 (3), e57841. 10.1371/journal.pone.0057841 23472110 PMC3589476

[B100] Leng Y. Chen Z. Ding H. Zhao X. Qin L. Pan Y. (2021). Overexpression of microRNA-29b Inhibits Epithelial-Mesenchymal Transition and Angiogenesis of Colorectal Cancer through the ETV4/ERK/EGFR axis. Cancer Cel Int 21 (1), 17. 10.1186/s12935-020-01700-2 PMC7789299 33407520

[B101] Leone P. Buonavoglia A. Fasano R. Solimando A. G. De Re V. Cicco S. (2019). Insights into the Regulation of Tumor Angiogenesis by Micro-RNAs. Jcm 8 (12), 2030. 10.3390/jcm8122030 PMC6947031 31757094

[B102] Lewis B. P. Burge C. B. Bartel D. P. (2005). Conserved Seed Pairing, Often Flanked by Adenosines, Indicates that Thousands of Human Genes Are microRNA Targets. Cell 120 (1), 15–20. 10.1016/j.cell.2004.12.035 15652477

[B103] Li J.-T. Wang L.-F. Zhao Y.-L. Yang T. Li W. Zhao J. (2014). RETRACTED ARTICLE: Nuclear Factor of Activated T Cells 5 Maintained by Hotair Suppression of miR-568 Upregulates S100 Calcium Binding Protein A4 to Promote Breast Cancer Metastasis. Breast Cancer Res. 16 (5), 454. 10.1186/s13058-014-0454-2 25311085 PMC4303133

[B104] Li J.-Y. Zhang Y. Zhang W.-H. Jia S. Kang Y. Zhu X.-Y. (2012). Differential Distribution of miR-20a and miR-20b May Underly Metastatic Heterogeneity of Breast Cancers. Asian Pac. J. Cancer Prev. 13 (5), 1901–1906. 10.7314/apjcp.2012.13.5.1901 22901144

[B105] Li J. Zhang C. Jiang H. Cheng J. (2015). Andrographolide Inhibits Hypoxia-Inducible Factor-1 through Phosphatidylinositol 3-kinase/AKT Pathway and Suppresses Breast Cancer Growth. Ott 8, 427–435. 10.2147/ott.s76116 PMC4335622 25709476

[B106] Li S. Mai H. Zhu Y. Li G. Sun J. Li G. (2020). MicroRNA-4500 Inhibits Migration, Invasion, and Angiogenesis of Breast Cancer Cells via RRM2-dependent MAPK Signaling Pathway. Mol. Ther. - Nucleic Acids 21, 278–289. 10.1016/j.omtn.2020.04.018 32615527 PMC7330432

[B107] Li Y. Cai B. Shen L. Dong Y. Lu Q. Sun S. (2017). MiRNA-29b Suppresses Tumor Growth through Simultaneously Inhibiting Angiogenesis and Tumorigenesis by Targeting Akt3. Cancer Lett. 397, 111–119. 10.1016/j.canlet.2017.03.032 28365400

[B108] Li Y. Zeng Q. a. Qiu J. Pang T. Ye F. Huang L. (2020). MiR-183-5p Promotes Proliferation, Metastasis and Angiogenesis in Breast Cancer Cells through Negatively Regulating Four and a Half LIM Protein 1. J. Breast Cancer 23 (4), 355–372. 10.4048/jbc.2020.23.e47 32908787 PMC7462817

[B109] Liang H. Ge F. Xu Y. Xiao J. Zhou Z. Liu R. (2018). miR-153 Inhibits the Migration and the Tube Formation of Endothelial Cells by Blocking the Paracrine of Angiopoietin 1 in Breast Cancer Cells. Angiogenesis 21 (4), 849–860. 10.1007/s10456-018-9630-9 29959560 PMC6208884

[B110] Liang H. Xiao J. Zhou Z. Wu J. Ge F. Li Z. (2018). Hypoxia Induces miR-153 through the IRE1α-XBP1 Pathway to fine Tune the HIF1α/VEGFA axis in Breast Cancer Angiogenesis. Oncogene 37 (15), 1961–1975. 10.1038/s41388-017-0089-8 29367761 PMC5895606

[B111] Liang L. Zhao L. Zan Y. Zhu Q. Ren J. Zhao X. (2017). MiR-93-5p Enhances Growth and Angiogenesis Capacity of HUVECs by Down-Regulating EPLIN. Oncotarget 8 (63), 107033–107043. 10.18632/oncotarget.22300 29291009 PMC5739794

[B112] Liang Z. Bian X. Shim H. (2016). Downregulation of microRNA-206 Promotes Invasion and Angiogenesis of Triple Negative Breast Cancer. Biochem. biophysical Res. Commun. 477 (3), 461–466. 10.1016/j.bbrc.2016.06.076 27318091

[B113] Lin J. Teo S. Lam D. H. Jeyaseelan K. Wang S. (2012). MicroRNA-10b Pleiotropically Regulates Invasion, Angiogenicity and Apoptosis of Tumor Cells Resembling Mesenchymal Subtype of Glioblastoma Multiforme. Cel Death Dis 3 (10), e398. 10.1038/cddis.2012.134 PMC3481123 23034333

[B114] Lin X. Qiu W. Xiao Y. Ma J. Xu F. Zhang K. (2019). MiR-199b-5p Suppresses Tumor Angiogenesis Mediated by Vascular Endothelial Cells in Breast Cancer by Targeting ALK1. Front. Genet. 10, 1397. 10.3389/fgene.2019.01397 32082362 PMC7002562

[B115] Lin X. Qiu W. Xiao Y. Ma J. Xu F. Zhang K. (2020). MiR-199b-5p Suppresses Tumor Angiogenesis Mediated by Vascular Endothelial Cells in Breast Cancer by Targeting ALK1. Front. Genet. 10, 1397. 10.3389/fgene.2019.01397 32082362 PMC7002562

[B116] Liu C. Duan P. Li B. Huang C. Jing Y. Yan W. (2015). miR-29a Activates Hes1 by Targeting Nfia in Esophageal Carcinoma Cell Line TE-1. Oncol. Lett. 9 (1), 96–102. 10.3892/ol.2014.2678 25435940 PMC4246642

[B117] Liu J. Zhou Y. Shi Z. Hu Y. Meng T. Zhang X. (2016). microRNA-497 Modulates Breast Cancer Cell Proliferation, Invasion, and Survival by Targeting SMAD7. DNA Cel Biol. 35 (9), 521–529. 10.1089/dna.2016.3282 27303812

[B118] Liu X. Guan Y. Wang L. Niu Y. (2017). MicroRNA-10b Expression in Node-Negative Breast Cancer-Correlation with Metastasis and Angiogenesis. Oncol. Lett. 14 (5), 5845–5852. 10.3892/ol.2017.6914 29113216 PMC5661387

[B119] Liu Y. Kong X. Li X. Li B. Yang Q. (2015). Knockdown of Metadherin Inhibits Angiogenesis in Breast Cancer. Int. J. Oncol. 46 (6), 2459–2466. 10.3892/ijo.2015.2973 25902416

[B120] Long H.-C. Gao X. Lei C.-J. Zhu B. Li L. Zeng C. (2016). miR-542-3p Inhibits the Growth and Invasion of Colorectal Cancer Cells through Targeted Regulation of Cortactin. Int. J. Mol. Med. 37 (4), 1112–1118. 10.3892/ijmm.2016.2505 26952924

[B121] Lu Y. Y. Sweredoski M. J. Huss D. Lansford R. Hess S. Tirrell D. A. (2014). Prometastatic GPCR CD97 Is a Direct Target of Tumor Suppressor microRNA-126. ACS Chem. Biol. 9 (2), 334–338. 10.1021/cb400704n 24274104 PMC3944050

[B122] Luengo-Gil G. Gonzalez-Billalabeitia E. Perez-Henarejos S. A. Navarro Manzano E. Chaves-Benito A. Garcia-Martinez E. (2018). Angiogenic Role of miR-20a in Breast Cancer. PLoS One 13 (4), e0194638. 10.1371/journal.pone.0194638 29617404 PMC5884522

[B123] Luo M. Tan X. Mu L. Luo Y. Li R. Deng X. (2017). MiRNA-21 Mediates the Antiangiogenic Activity of Metformin through Targeting PTEN and SMAD7 Expression and PI3K/AKT Pathway. Sci. Rep. 7, 43427. 10.1038/srep43427 28230206 PMC5322530

[B124] Luo Q. Wang J. Zhao W. Peng Z. Liu X. Li B. (2020). Vasculogenic Mimicry in Carcinogenesis and Clinical Applications. J. Hematol. Oncol. 13 (1), 19. 10.1186/s13045-020-00858-6 32169087 PMC7071697

[B125] Lyu H. Wang S. Huang J. Wang B. He Z. Liu B. (2018). Survivin -targeting miR-542-3p Overcomes HER3 Signaling-Induced Chemoresistance and Enhances the Antitumor Activity of Paclitaxel against HER2-Overexpressing Breast Cancer. Cancer Lett. 420, 97–108. 10.1016/j.canlet.2018.01.065 29409974 PMC6089084

[B126] Ma L. Young J. Prabhala H. Pan E. Mestdagh P. Muth D. (2010). miR-9, a MYC/MYCN-activated microRNA, Regulates E-Cadherin and Cancer Metastasis. Nat. Cel Biol 12 (3), 247–256. 10.1038/ncb2024 PMC2845545 20173740

[B127] Maeshima Y. Sudhakar A. Lively J. C. Ueki K. Kharbanda S. Kahn C. R. (2002). Tumstatin, an Endothelial Cell-specific Inhibitor of Protein Synthesis. Science 295 (5552), 140–143. 10.1126/science.1065298 11778052

[B128] Magnon C. Galaup A. Mullan B. Rouffiac V. Bidart J.-M. Griscelli F. (2005). Canstatin Acts on Endothelial and Tumor Cells via Mitochondrial Damage Initiated through Interaction with αvβ3 and αvβ5 Integrins. Cancer Res. 65 (10), 4353–4361. 10.1158/0008-5472.can-04-3536 15899827

[B129] Magura J. Moodley R. Mackraj I. (2020). The Effect of Hesperidin and Luteolin Isolated from Eriocephalus Africanus on Apoptosis, Cell Cycle and miRNA Expression in MCF-7. J. Biomol. Struct. Dyn., 1–10. 10.1080/07391102.2020.1833757 33050842

[B130] Majumder M. Dunn L. Liu L. Hasan A. Vincent K. Brackstone M. (2018). COX-2 Induces Oncogenic Micro RNA miR655 in Human Breast Cancer. Sci. Rep. 8 (1), 327. 10.1038/s41598-017-18612-3 29321644 PMC5762661

[B131] Majumder M. Landman E. Liu L. Hess D. Lala P. K. (2015). COX-2 Elevates Oncogenic miR-526b in Breast Cancer by EP4 Activation. Mol. Cancer Res. 13 (6), 1022–1033. 10.1158/1541-7786.mcr-14-0543 25733698

[B132] Maroufi N. F. Amiri M. Dizaji B. F. Vahedian V. Akbarzadeh M. Roshanravan N. (2020). Inhibitory Effect of Melatonin on Hypoxia-Induced Vasculogenic Mimicry via Suppressing Epithelial-Mesenchymal Transition (EMT) in Breast Cancer Stem Cells. Eur. J. Pharmacol. 881, 173282. 10.1016/j.ejphar.2020.173282 32580038

[B133] Marques J. H. M. Mota A. L. Oliveira J. G. Lacerda J. Z. Stefani J. P. Ferreira L. C. (2018). Melatonin Restrains Angiogenic Factors in Triple-Negative Breast Cancer by Targeting miR-152-3p: *In Vivo* and *In Vitro* Studies. Life Sci. 208, 131–138. 10.1016/j.lfs.2018.07.012 29990486

[B134] Martello G. Rosato A. Ferrari F. Manfrin A. Cordenonsi M. Dupont S. (2010). A MicroRNA Targeting Dicer for Metastasis Control. Cell 141 (7), 1195–1207. 10.1016/j.cell.2010.05.017 20603000

[B135] Mertens-Talcott S. U. Noratto G. D. Li X. Angel-Morales G. Bertoldi M. C. Safe S. (2013). Betulinic Acid Decreases ER-Negative Breast Cancer Cell Growth *In Vitro* and *In Vivo*: Role of Sp Transcription Factors and microRNA-27a:ZBTB10. Mol. Carcinog. 52 (8), 591–602. 10.1002/mc.21893 22407812 PMC3418350

[B136] Mirzaaghaei S. Foroughmand A. M. Saki G. Shafiei M. (2019). Combination of Epigallocatechin-3-Gallate and Silibinin: A Novel Approach for Targeting Both Tumor and Endothelial Cells. ACS Omega 4 (5), 8421–8430. 10.1021/acsomega.9b00224 31459931 PMC6648523

[B137] Mu J. Zhu D. Shen Z. Ning S. Liu Y. Chen J. (2017). The Repressive Effect of miR-148a on Wnt/β-Catenin Signaling Involved in Glabridin-Induced Anti-angiogenesis in Human Breast Cancer Cells. BMC Cancer 17 (1), 307. 10.1186/s12885-017-3298-1 28464803 PMC5414299

[B138] Nakamura Y. Patrushev N. Inomata H. Mehta D. Urao N. Kim H. W. (2008). Role of Protein Tyrosine Phosphatase 1B in Vascular Endothelial Growth Factor Signaling and Cell-Cell Adhesions in Endothelial Cells. Circ. Res. 102 (10), 1182–1191. 10.1161/circresaha.107.167080 18451337 PMC2737681

[B139] Norouzi S. Majeed M. Pirro M. Generali D. Sahebkar A. (2018). Curcumin as an Adjunct Therapy and microRNA Modulator in Breast Cancer. Cpd 24 (2), 171–177. 10.2174/1381612824666171129203506 29189128

[B140] O'Reilly M. S. Boehm T. Shing Y. Fukai N. Vasios G. Lane W. S. (1997). Endostatin: an Endogenous Inhibitor of Angiogenesis and Tumor Growth. Cell 88 (2), 277–285. 10.1016/s0092-8674(00)81848-6 9008168

[B141] Oliveras-Ferraros C. Cufí S. Vazquez-Martin A. Torres-Garcia V. Z. Del Barco S. Martin-Castillo B. (2011). Micro(mi)RNA Expression Profile of Breast Cancer Epithelial Cells Treated with the Anti-diabetic Drug Metformin: Induction of the Tumor Suppressor miRNA Let-7a and Suppression of the TGFβ-Induced oncomiR miRNA-181a. Cell Cycle 10 (7), 1144–1151. 10.4161/cc.10.7.15210 21368581

[B142] Pakravan K. Babashah S. Sadeghizadeh M. Mowla S. J. Mossahebi-Mohammadi M. Ataei F. (2017). MicroRNA-100 Shuttled by Mesenchymal Stem Cell-Derived Exosomes Suppresses *In Vitro* Angiogenesis through Modulating the mTOR/HIF-1α/VEGF Signaling axis in Breast Cancer Cells. Cell Oncol. 40 (5), 457–470. 10.1007/s13402-017-0335-7 PMC13001539 28741069

[B143] Pan L. Huang S. He R. Rong M. Dang Y. Chen G. (2014). Decreased Expression and Clinical Significance of miR-148a in Hepatocellular Carcinoma Tissues. Eur. J. Med. Res. 19 (1), 68. 10.1186/s40001-014-0068-2 25444499 PMC4258268

[B144] Pan S. Zhao X. Shao C. Fu B. Huang Y. Zhang N. (2021). STIM1 Promotes Angiogenesis by Reducing Exosomal miR-145 in Breast Cancer MDA-MB-231 Cells. Cel Death Dis 12 (1), 38. 10.1038/s41419-020-03304-0 PMC7791041 33414420

[B145] Panahi G. (2018). The Effects of Quercetin on miRNA-21 Expression in MCF-7 Cells. Arch. Med. Lab. Sci. 3 (3). 10.22037/amls.v3i3.21696

[B146] Pecot C. V. Rupaimoole R. Yang D. Akbani R. Ivan C. Lu C. (2013). Tumour Angiogenesis Regulation by the miR-200 Family. Nat. Commun. 4 (1), 2427. 10.1038/ncomms3427 24018975 PMC3904438

[B147] Pei Y.-f. Lei Y. Liu X.-q. (2016). MiR-29a Promotes Cell Proliferation and EMT in Breast Cancer by Targeting Ten Eleven Translocation 1. Biochim. Biophys. Acta (Bba) - Mol. Basis Dis. 1862 (11), 2177–2185. 10.1016/j.bbadis.2016.08.014 27555295

[B148] Plummer P. N. Freeman R. Taft R. J. Vider J. Sax M. Umer B. A. (2013). MicroRNAs Regulate Tumor Angiogenesis Modulated by Endothelial Progenitor Cells. Cancer Res. 73 (1), 341–352. 10.1158/0008-5472.can-12-0271 22836757

[B149] Png K. J. Halberg N. Yoshida M. Tavazoie S. F. (2011). A microRNA Regulon that Mediates Endothelial Recruitment and Metastasis by Cancer Cells. Nature 481 (7380), 190–194. 10.1038/nature10661 22170610

[B150] Poliseno L. Tuccoli A. Mariani L. Evangelista M. Citti L. Woods K. (2006). MicroRNAs Modulate the Angiogenic Properties of HUVECs. Blood 108 (9), 3068–3071. 10.1182/blood-2006-01-012369 16849646

[B151] Pulito C. Mori F. Sacconi A. Goeman F. Ferraiuolo M. Pasanisi P. (2017). Metformin-induced Ablation of microRNA 21-5p Releases Sestrin-1 and CAB39L Antitumoral Activities. Cell Discov 3, 17022. 10.1038/celldisc.2017.22 28698800 PMC5501975

[B152] Qiu T. Y. Huang J. Wang L. P. Zhu B. S. (2021). Inhibition of miR-200b Promotes Angiogenesis in Endothelial Cells by Activating the Notch Pathway. Cell J 23 (1), 51–60. 10.22074/cellj.2021.7080 33650820 PMC7944128

[B153] Rostas J. W. 3rd Pruitt H. C. Metge B. J. Mitra A. Bailey S. K. Bae S. (2014). microRNA-29 Negatively Regulates EMT Regulator N-Myc Interactor in Breast Cancer. Mol. Cancer 13, 200. 10.1186/1476-4598-13-200 25174825 PMC4169820

[B154] Salgado E. Bian X. Feng A. Shim H. Liang Z. (2018). HDAC9 Overexpression Confers Invasive and Angiogenic Potential to Triple Negative Breast Cancer Cells via Modulating microRNA-206. Biochem. Biophysical Res. Commun. 503 (2), 1087–1091. 10.1016/j.bbrc.2018.06.120 PMC6439468 29936177

[B155] Salinas-Vera Y. M. Marchat L. A. Gallardo-Rincón D. Ruiz-García E. Astudillo-De La Vega H. Echavarría-Zepeda R. (2019). AngiomiRs: MicroRNAs Driving Angiogenesis in Cancer (Review). Int. J. Mol. Med. 43 (2), 657–670. 10.3892/ijmm.2018.4003 30483765

[B156] Salinas-Vera Y. M. Marchat L. A. García-Vázquez R. González de la Rosa C. H. Castañeda-Saucedo E. Tito N. N. (2018). Cooperative Multi-Targeting of Signaling Networks by angiomiR-204 Inhibits Vasculogenic Mimicry in Breast Cancer Cells. Cancer Lett. 432, 17–27. 10.1016/j.canlet.2018.06.003 29885516

[B157] Semenza G. L. (2003). Targeting HIF-1 for Cancer Therapy. Nat. Rev. Cancer 3 (10), 721–732. 10.1038/nrc1187 13130303

[B158] Sharma P. Kumar S. (2018). Metformin Inhibits Human Breast Cancer Cell Growth by Promoting Apoptosis via a ROS-independent Pathway Involving Mitochondrial Dysfunction: Pivotal Role of Superoxide Dismutase (SOD). Cel Oncol. 41 (6), 637–650. 10.1007/s13402-018-0398-0 PMC12995255 30088260

[B159] Shen X. Fang J. Lv X. Pei Z. Wang Y. Jiang S. (2011). Heparin Impairs Angiogenesis through Inhibition of microRNA-10b. J. Biol. Chem. 286 (30), 26616–26627. 10.1074/jbc.m111.224212 21642433 PMC3143626

[B160] Siegel R. L. Miller K. D. Fuchs H. E. Jemal A. (2021). Cancer Statistics, 2021. CA A. Cancer J. Clin. 71 (1), 7–33. 10.3322/caac.21654 33433946

[B161] Siemann D. W. (2011). The Unique Characteristics of Tumor Vasculature and Preclinical Evidence for its Selective Disruption by Tumor-Vascular Disrupting Agents. Cancer Treat. Rev. 37 (1), 63–74. 10.1016/j.ctrv.2010.05.001 20570444 PMC2958232

[B162] Singh R. Yadav V. Kumar S. Saini N. (2015). MicroRNA-195 Inhibits Proliferation, Invasion and Metastasis in Breast Cancer Cells by Targeting FASN, HMGCR, ACACA and CYP27B1. Sci. Rep. 5, 17454. 10.1038/srep17454 26632252 PMC4668367

[B163] Sobhkhizi A. Babaei E. Azeez H. J. Katiraee F. Hussen B. M. Hoseinpour Feizi M. A. (2020). Dendrosomal Nano-Curcumin Modulates P-Glycoprotein Activity and Induces Apoptosis in Wild Type and P53-Mutant Breast Cancer Cell Lines. Jentashapir J. Cel Mol Biol 11 (4), e109143. 10.5812/jjcmb.109143

[B164] Soheilifar M. H. Masoudi-Khoram N. Madadi S. Nobari S. Maadi H. Keshmiri Neghab H. (2021). Angioregulatory microRNAs in Breast Cancer: Molecular Mechanistic Basis and Implications for Therapeutic Strategies. J. Adv. Res. 10.1016/j.jare.2021.06.019 PMC9039675 35499045

[B165] Soung Y. H. Korneeva N. Kim T. H. Chung J. (2013). The Role of C-Src in Integrin (α6β4) Dependent Translational Control. BMC Cel Biol 14, 49. 10.1186/1471-2121-14-49 PMC4228388 24180592

[B166] Suárez Y. Fernández-Hernando C. Pober J. S. Sessa W. C. (2007). Dicer Dependent microRNAs Regulate Gene Expression and Functions in Human Endothelial Cells. Circ. Res. 100 (8), 1164–1173. 10.1161/01.RES.0000265065.26744.17 17379831

[B167] Suárez Y. Sessa W. C. (2009). MicroRNAs as Novel Regulators of Angiogenesis. Circ. Res. 104 (4), 442–454. 10.1161/CIRCRESAHA.108.191270 19246688 PMC2760389

[B168] Sugio A. Iwasaki M. Habata S. Mariya T. Suzuki M. Osogami H. (2014). BAG3 Upregulates Mcl-1 through Downregulation of miR-29b to Induce Anticancer Drug Resistance in Ovarian Cancer. Gynecol. Oncol. 134 (3), 615–623. 10.1016/j.ygyno.2014.06.024 24992675

[B169] Sun G. Liu M. Han H. (2019). Overexpression of microRNA‐190 Inhibits Migration, Invasion, Epithelial‐mesenchymal Transition, and Angiogenesis through Suppression of Protein Kinase B‐extracellular Signal‐regulated Kinase Signaling Pathway via Binding to Stanniocalicin 2 in Breast Cancer. J. Cel Physiol 234 (10), 17824–17838. 10.1002/jcp.28409 30993707

[B170] Sun L. L. Li W. D. Lei F. R. Li X. Q. (2018). The Regulatory Role of Micro RNA S in Angiogenesis‐related Diseases. J. Cel Mol Med 22 (10), 4568–4587. 10.1111/jcmm.13700 PMC6156236 29956461

[B171] Taheri M. Mahmud Hussen B. Tondro Anamag F. Shoorei H. Dinger M. E. Ghafouri-Fard S. (2021). The Role of miRNAs and lncRNAs in Conferring Resistance to Doxorubicin. J. Drug Target., 1–21. 10.1080/1061186x.2021.1909052 33788650

[B172] Talcott S. U. Li X. Chintharlapalli S. Safe S. (2008). The Effects of Betulinic Acid on microRNA‐27a Regulated Target Genes in MDA‐MB‐231 Breast Cancer Cells. Wiley Online Library.

[B173] Tanimoto K. Makino Y. Pereira T. Poellinger L. (2000). Mechanism of regulation of the hypoxia-inducible factor-1alpha by the von Hippel-Lindau tumor suppressor protein. EMBO J. 19 (16), 4298–4309. 10.1093/emboj/19.16.4298 10944113 PMC302039

[B174] Tehler D. Høyland-Kroghsbo N. M. Lund A. H. (2011). The miR-10 microRNA Precursor Family. RNA Biol. 8 (5), 728–734. 10.4161/rna.8.5.16324 21881411 PMC3256350

[B175] Tu Y. Liu L. Zhao D. Liu Y. Ma X. Fan Y. (2015). Overexpression of miRNA-497 Inhibits Tumor Angiogenesis by Targeting VEGFR2. Sci. Rep. 5, 13827. 10.1038/srep13827 26345385 PMC4561885

[B176] Varghese E. Liskova A. Kubatka P. Samuel S. M. Büsselberg D. (2020). Anti-Angiogenic Effects of Phytochemicals on miRNA Regulating Breast Cancer Progression. Biomolecules 10 (2), 191. 10.3390/biom10020191 PMC7072640 32012744

[B177] Vasudevan S. Tong Y. Steitz J. A. (2007). Switching from Repression to Activation: microRNAs Can Up-Regulate Translation. Science 318 (5858), 1931–1934. 10.1126/science.1149460 18048652

[B178] Wang J. Li G. Wang Y. Tang S. Sun X. Feng X. (2015). Suppression of Tumor Angiogenesis by Metformin Treatmentviaa Mechanism Linked to Targeting of HER2/HIF-1α/VEGF Secretion axis. Oncotarget 6 (42), 44579–44592. 10.18632/oncotarget.6373 26625311 PMC4792577

[B179] Wang W. Dong J. Wang M. Yao S. Tian X. Cui X. (2018). miR-148a-3p Suppresses Epithelial Ovarian Cancer Progression Primarily by Targeting C-Met. Oncol. Lett. 15 (5), 6131–6136. 10.3892/ol.2018.8110 29616095 PMC5876423

[B180] Wang X.-P. Yao J. Guan J. Zhou Z.-Q. Zhang Z.-Y. Yang J. (2018). MicroRNA-542-3p Functions as a Tumor Suppressor via Directly Targeting Survivin in Hepatocellular Carcinoma. Biomed. Pharmacother. 99, 817–824. 10.1016/j.biopha.2018.01.131 29710480

[B181] Wang X. Hang Y. Liu J. Hou Y. Wang N. Wang M. (2017). Anticancer Effect of Curcumin Inhibits Cell Growth through miR-21/PTEN/Akt Pathway in Breast Cancer Cell. Oncol. Lett. 13 (6), 4825–4831. 10.3892/ol.2017.6053 28599484 PMC5452995

[B182] Wang Y. Wang L. Chen C. Chu X. (2018). New Insights into the Regulatory Role of microRNA in Tumor Angiogenesis and Clinical Implications. Mol. Cancer 17 (1), 22. 10.1186/s12943-018-0766-4 29415727 PMC5804051

[B183] Wang Y. Zhang X. Zou C. Kung H.-F. Lin M. C. Dress A. (2016). miR-195 Inhibits Tumor Growth and Angiogenesis through Modulating IRS1 in Breast Cancer. Biomed. Pharmacother. 80, 95–101. 10.1016/j.biopha.2016.03.007 27133044

[B184] Wei X. Chen Y. Jiang X. Peng M. Liu Y. Mo Y. (2021). Mechanisms of Vasculogenic Mimicry in Hypoxic Tumor Microenvironments. Mol. Cancer 20 (1), 7. 10.1186/s12943-020-01288-1 33397409 PMC7784348

[B185] Wu K. He J. Pu W. Peng Y. (2018). The Role of Exportin-5 in MicroRNA Biogenesis and Cancer. Genomics, Proteomics & Bioinformatics 16 (2), 120–126. 10.1016/j.gpb.2017.09.004 PMC6112314 29723684

[B186] Wu M.-Z. Cheng W.-C. Chen S.-F. Nieh S. O’Connor C. Liu C.-L. (2017). miR-25/93 Mediates Hypoxia-Induced Immunosuppression by Repressing cGAS. Nat. Cel Biol 19 (10), 1286–1296. 10.1038/ncb3615 PMC5658024 28920955

[B187] Wu Z. Cai X. Huang C. Xu J. Liu A. (2016). miR-497 Suppresses Angiogenesis in Breast Carcinoma by Targeting HIF-1α. Oncol. Rep. 35 (3), 1696–1702. 10.3892/or.2015.4529 26718330

[B188] Xu Q. Jiang Y. Yin Y. Li Q. He J. Jing Y. (2012). A Regulatory Circuit of miR-148a/152 and DNMT1 in Modulating Cell Transformation and Tumor Angiogenesis through IGF-IR and IRS1. J. Mol. Cel Biol. 5 (1), 3–13. 10.1093/jmcb/mjs049 PMC3570052 22935141

[B189] Xu Q. Jiang Y. Yin Y. Li Q. He J. Jing Y. (2013). A Regulatory Circuit of miR-148a/152 and DNMT1 in Modulating Cell Transformation and Tumor Angiogenesis through IGF-IR and IRS1. J. Mol. Cel Biol. 5 (1), 3–13. 10.1093/jmcb/mjs049 PMC3570052 22935141

[B190] Yan L.-X. Huang X.-F. Shao Q. Huang M.-Y. Deng L. Wu Q.-L. (2008). MicroRNA miR-21 Overexpression in Human Breast Cancer Is Associated with Advanced Clinical Stage, Lymph Node Metastasis and Patient Poor Prognosis. RNA 14 (11), 2348–2360. 10.1261/rna.1034808 18812439 PMC2578865

[B191] Yang J. Cao Y. Sun J. Zhang Y. (2010). Curcumin Reduces the Expression of Bcl-2 by Upregulating miR-15a and miR-16 in MCF-7 Cells. Med. Oncol. 27 (4), 1114–1118. 10.1007/s12032-009-9344-3 19908170

[B192] Yang J. P. Liao Y. D. Mai D. M. Xie P. Qiang Y. Y. Zheng L. S. (2016). Tumor Vasculogenic Mimicry Predicts Poor Prognosis in Cancer Patients: a Meta-Analysis. Angiogenesis 19 (2), 191–200. 10.1007/s10456-016-9500-2 26899730

[B193] Yin R. Guo L. Gu J. Li C. Zhang W. (2018). Over Expressing miR-19b-1 Suppress Breast Cancer Growth by Inhibiting Tumor Microenvironment Induced Angiogenesis. Int. J. Biochem. Cel Biol. 97, 43–51. 10.1016/j.biocel.2018.02.005 29425833

[B194] Yu J. Li Q. Xu Q. Liu L. Jiang B. (2011). MiR-148a Inhibits Angiogenesis by Targeting ERBB3. J. Biomed. Res. 25 (3), 170–177. 10.1016/s1674-8301(11)60022-5 23554686 PMC3597061

[B195] Yu W. Liang X. Li X. Zhang Y. Sun Z. Liu Y. (2018). MicroRNA-195: a Review of its Role in Cancers. Ott 11, 7109–7123. 10.2147/ott.s183600 PMC6200091 30410367

[B196] Yu W. Yang L. Li T. Zhang Y. (2019). Cadherin Signaling in Cancer: Its Functions and Role as a Therapeutic Target. Front. Oncol. 9, 989. 10.3389/fonc.2019.00989 31637214 PMC6788064

[B197] Zadeh M. M. Ranji N. Motamed N. (2015). Deregulation of miR-21 and miR-155 and Their Putative Targets after Silibinin Treatment in T47D Breast Cancer Cells. Iran J. Basic Med. Sci. 18 (12), 1209–1214. 26877850 PMC4744360

[B198] Zeng Y. Cullen B. R. (2006). Recognition and Cleavage of Primary microRNA Transcripts. Methods Mol. Biol. 342, 49–56. 10.1385/1-59745-123-1:49 16957366

[B199] Zhang J. Li G. Chen Y. Fang L. Guan C. Bai F. (2017). Metformin Inhibits Tumorigenesis and Tumor Growth of Breast Cancer Cells by Upregulating miR-200c but Downregulating AKT2 Expression. J. Cancer 8 (10), 1849–1864. 10.7150/jca.19858 28819383 PMC5556649

[B200] Zhao D. Tu Y. Wan L. Bu L. Huang T. Sun X. (2013). *In Vivo* monitoring of Angiogenesis Inhibition via Down-Regulation of Mir-21 in a VEGFR2-Luc Murine Breast Cancer Model Using Bioluminescent Imaging. PLoS One 8 (8), e71472. 10.1371/journal.pone.0071472 23951172 PMC3738509

[B201] Zhou Q. Anderson C. Hanus J. Zhao F. Ma J. Yoshimura A. (2016). Strand and Cell Type-specific Function of microRNA-126 in Angiogenesis. Mol. Ther. 24 (10), 1823–1835. 10.1038/mt.2016.108 27203443 PMC5112035

[B202] Zhou W. Shi G. Zhang Q. Wu Q. Li B. Zhang Z. (2014). MicroRNA-20b Promotes Cell Growth of Breast Cancer Cells Partly via Targeting Phosphatase and Tensin Homologue (PTEN). Cell Biosci 4 (1), 62. 10.1186/2045-3701-4-62 25364498 PMC4216355

[B203] Zhu N. Zhang D. Xie H. Zhou Z. Chen H. Hu T. (2011). Endothelial-specific Intron-Derived miR-126 Is Down-Regulated in Human Breast Cancer and Targets Both VEGFA and PIK3R2. Mol. Cel Biochem 351 (1-2), 157–164. 10.1007/s11010-011-0723-7 21249429

